# Assessing climate change-robustness of protected area management plans—The case of Germany

**DOI:** 10.1371/journal.pone.0185972

**Published:** 2017-10-05

**Authors:** Juliane Geyer, Stefan Kreft, Florian Jeltsch, Pierre L. Ibisch

**Affiliations:** 1 Centre for Econics and Ecosystem Management, Faculty of Forest and Environment, Eberswalde University for Sustainable Development, Eberswalde, Germany; 2 Institute of Biochemistry and Biology, Potsdam University, Potsdam, Germany; University of Colorado, UNITED STATES

## Abstract

Protected areas are arguably the most important instrument of biodiversity conservation. To keep them fit under climate change, their management needs to be adapted to address related direct and indirect changes. In our study we focus on the adaptation of conservation management planning, evaluating management plans of 60 protected areas throughout Germany with regard to their climate change-robustness. First, climate change-robust conservation management was defined using 11 principles and 44 criteria, which followed an approach similar to sustainability standards. We then evaluated the performance of individual management plans concerning the climate change-robustness framework. We found that climate change-robustness of protected areas hardly exceeded 50 percent of the potential performance, with most plans ranking in the lower quarter. Most Natura 2000 protected areas, established under conservation legislation of the European Union, belong to the sites with especially poor performance, with lower values in smaller areas. In general, the individual principles showed very different rates of accordance with our principles, but similarly low intensity. Principles with generally higher performance values included holistic knowledge management, public accountability and acceptance as well as systemic and strategic coherence. Deficiencies were connected to dealing with the future and uncertainty. Lastly, we recommended the presented principles and criteria as essential guideposts that can be used as a checklist for working towards more climate change-robust planning.

## Introduction

Conservation has long been confronted with the increasing impacts of climate change and the need to adapt to it. Protected areas are considered the cornerstones of conservation strategies and must also adapt to climate change while continuing to conserve biodiversity [1–5]. On one hand, protected areas are vulnerable to climate change because they are often conceptualised statically, despite the spatially and temporally dynamic nature of their conservation objects (e.g. individual species, communities or ecosystems) [1, 6–8]. Nonetheless, they provide an insurance policy for biodiversity as refuges for ecosystem recovery and for preserving systems that are sensitive to disturbance [9]. Protected areas can also promote the adaptive capacity of ecosystems by providing a buffer against the rate and intensity of environmental change, or providing habitat corridors to facilitate species range- or ecosystem regime- shifts [10–12]. In general, major conservation organizations argue that protected areas are the best means of addressing climate change since they play a significant role in both mitigation and adaptation [13, 14]. Additionally, they are supposed to be places of high ecological integrity and functionality, which more or less embrace sustainable, organized and effective land use management [14].

It is due to this particular standing that protected areas, especially large-scale conservation sites, should take up a leading role not only for traditional biodiversity conservation but also as potential cores for the crystallization of integrative conservation in the wider landscape [15]. Thus, they may serve as catalysts for the implementation of the principles of ecosystem-based management and ecosystem-based adaptation (e.g., the Ecosystem Approach [16] in [15, 17]). However, protected areas can only take up this role if their vulnerability to climate change can effectively be reduced. A considerable number of publications with general recommendations on how to integrate climate change into conservation management and planning are now available [18–22]. Some studies and reports provide specific practical guidance for protected areas with real world examples [23–28].

There are different lines of action for conservation, and for protected areas in particular, to face climate change and reduce vulnerability. One is to tackle the vulnerability of conservation targets or biodiversity objects, i.e. reduce the biotic vulnerability [29, 30]. This approach is prevalent in existing literature (e.g., [18, 19, 31]). The majority of conservation action associated with this approach depends on the regional or local specifics of the conservation system and its influencing factors. Nevertheless, it is possible to extract generally valid principles. These include reducing threats not related to climate change, ecological restoration, assisted migration or enhancing connectivity, e.g. by the expansion of the reserve network [3, 18, 19, 29].

Thus, most recommendations found in the existing literature focus more or less on direct interventions with biodiversity. However, one part of the vulnerability of protected areas is also caused by human interactions within and with the protected area and its human non-human parts [32]. Nevertheless, options to reduce the vulnerability of conservation management on the levels of planning and implementation procedures are less often considered. A more holistic approach will therefore include all decisions and actions that directly or indirectly influence goal achievement of a conservation site [33]. Many of those planning and procedural options can be considered climate change-robust (or no-regret) adaptation options [34–36]. This means they will strengthen conservation with regard to climate change irrespectively of the exact manifestation of change, or with regard to other environmental or political changes. These robust strategies may also improve the performance and effectiveness of conservation management even if no changes occur [29].

A considerable number of evaluations of protected area management effectiveness in general have been conducted applying various methods, most of which are based on the IUCN Management Effectiveness Framework (e.g., [37–39]) but also other approaches [40]. The contemporary evaluation criteria for protected area management effectiveness [37, 40–42] consider climate change merely as one out of many factors that need to be considered, for instance as a threat to biodiversity [37]. However, unlike many other conventional threats, climate change is an overarching multifaceted element affecting all parts of any conservation system unprecedentedly, including other threats and their underlying drivers, [43] as well as the management itself. Therefore, all components of a management system contribute to conservation effectiveness under climate change and need to be tailored specifically to this effect. This has been rarely reflected by research on protected area management. Evaluations of local climate change adaptation plans have been conducted without a specific focus on conservation, for example in the USA [44], in Australia [45], Canada [46] and India [47]. One recent study in Germany assessed to what degree conservation plans at the county level addressed potential impacts of climate change [48]. In a study on the vulnerability of German protected areas to climate change, Kreft et al. [33] also included, among others, some aspects of conservation management itself. They found that management-related issues were the most significant contributing factor influencing the vulnerability of protected areas to climate change in Germany.

Because of these findings, and to fill the knowledge gap regarding suitable evaluation criteria for climate change adaptation in protected areas, we present principles of climate change-robust management. Our objectives are to guide conservation planning and to provide a framework for assessing the quality and effectiveness of those plans. We define climate change-robustness in protected area management as conservation management that is effective in sustaining the functionality of a protected area despite the myriad of potential impacts associated with climate change. Climate change-robustness heavily builds upon a sturdy fundament of effective management combined with add-ons specifically tailored to tackling climate change. The presented principles hence elaborate on, rather than contradict, existing criteria for evaluating management effectiveness in addressing issues related to climate change. We use our proposed set of principles for climate change-robust conservation management in building an evaluation framework for assessing climate change-robustness of protected area management plans, focussing on a case study in Germany. Further, we analyse the accordance of protected areas and the respective management plans with individual robustness principles. Specifically, we address the following research questions:

1. How does management planning of protected areas perform with regard to climate change-robustness? How do different protected areas in Germany compare in their robustness?
2. How do individual principles and criteria of climate change-robustness perform across German protected areas? Which of these principles and criteria have received comparatively better attention, and which ones have so far been neglected?

## Methods

### Sample selection of management plans

Our study covers management plans from 60 protected areas in Germany: seven national parks (NLP), six biosphere reserves (BR), and 14 nature parks (NP) as well as 33 protected areas established under the conservation regime of the European Union, the so-called Natura 2000 sites (see Table 1 and S1 Text for a description of protected area categories and S2 Table for the list of management plans). We chose these categories because they can also be found outside Germany. Additionally, for NLP, BR and NP the preparation of a management plan is one essential criterion of the corresponding management quality standards in Germany [49–51]. For Natura 2000 sites the German Agency for Nature Conservation recommends the elaboration of management plans following the European Commission, suggesting management plans as one instrument for securing the conservation status of Natura 2000 sites [52, 53].

**Table 1:** Short description of the four protected area categories selected for the analysis (for more detailed description see S1 Text).

National Parks	Biosphere Reserves	Nature Parks	Natura 2000 sites
National parks serve to protect ecological processes and shall develop into areas free of human intervention [54].	Biosphere reserves are established according to the UNESCO Man and the Biosphere Programme; they integrate biodiversity conservation and exemplary sustainable land use in historically evolved cultural and natural landscapes [54].	Nature parks combine conservation with recreation and specifically support sustainable rural development [54].	Natura 2000 is a European network of protected areas aiming at the protection of vulnerable habitats and species listed under both the EU Birds Directive and the Habitats Directive across their natural range in Europe and ensure that they are restored to, or maintained at, a favourable conservation status [55].

In Germany, the administrations of the 16 federal states are responsible for conservation management. The different types of (national) protected areas are defined in Germany's Federal Nature Conservation Act. The large protected areas (*Großschutzgebiete*) together cover 30% of the country’s territory (partly overlapping with other protected areas and among themselves) [56]. Approximately 15% of German territory is covered by 5,266 protected sites of the European Natura 2000 network as reported under the EU Habitats Directive (4,621 Special Areas of Conservation—SAC) and Birds Directive (738 Special Protection Areas—SPA), but they are partly overlapping with national protected areas. The German Federal Agency for nature Conservation (BfN) recommends the elaboration of management plans for all Natura 2000 sites with participation of land users and stakeholders according to specific requirements compiled in a guideline [53, 57]. However, for numerous Natura 2000 sites management plans have not yet been completed [58].

For our analysis, we chose–for the four categories of protected areas–exclusively management plans, or those plans that currently have the function of a management plan, for the four categories of protected areas. This included documents called *Managementplan* (management plan), *Nationalparkplan* (national park plan), *Naturparkplan* (nature park plan), *Biosphärenreservatsplan* (biosphere reserve plan), *Rahmenkonzept* (conceptual framework), or different types of development plans such as, *Pflege- und Entwicklungsplan* (maintenance and development plan), *Pflege- und Entwicklungskonzept* (maintenance and development concept), *Pflege- und Managementplan* (maintenance and management plan), *Bewirtschaftungsplan* (management plan) and *Sofortmaßnahmenkonzept* (immediate action concept). It must be emphasized that we evaluated only the contents of the management plans and not their actual interpretation and implementation.

We selected plans created between 2003 and 2013 that were available online in pdf format. In cases where only short versions were available, we requested full versions from the responsible administration unit. Pre-versions or partially accomplished versions were excluded from the analysis. For BR and NLP we contacted the administrations separately if management plan status could not be determined. To acquire the management plans of the NP and Natura 2000 sites, we contacted several responsible institutions. However, the number of replies was very low, particularly for the latter sites. This resulted in unequal representation among federal states. We tried to choose plans of varying ages for each federal state. In the case of NP we pursued the guideline of one park per federal state as far as the availability and rate of reply allowed.

### Developing the evaluation protocol

We developed a protocol for the evaluation of the selected management plans in accordance with sustainability certification where principles and subordinated criteria are used to define sustainable practice (e.g., FSC [59] or RSPO [60]). The evaluation framework for our analysis was based on three types of sources regarding climate change adaptation strategies: 1) fixed standards for holistic, sustainable, and equitable natural resource and conservation management (such as the Ecosystem Approach), 2) scientific publications on options and recommendations for adapting conservation to climate change and 3) experience from project activities on management planning integrating climate change. From these, the most common and most conclusive recommendations were extracted (Table 2). Finally, we defined eleven principles of climate change-robust conservation management, and identified 44 guiding criteria (four for each principle) to enable assessment (Table 3, S1 Table). The majority of principles and criteria resemble those of best practice conservation management, with a new and/or stronger significance with regard to climate change. A more detailed description of the principles and their contribution to climate change-robustness of conservation management can be found in S1 Table. We applied an equal weighting approach to the criteria, which is the standard approach among the plan quality evaluation literature [61]. While this approach neglects potential differences among variables regarding their effects on index results [61–63], we chose it for its favourable transparency and interpretability in comparison to a weighted-factor approach.

**Table 2:** Main sources for defining principles and criteria of climate change-robustness used in the evaluation protocol (listed in chronological order).

Source (Content)	Year	Reference
Ecosystem Approach	2000	[16]
Systematic analysis of options of action for conservation to adapt to climate change	2008	[64]
List of recommendations for climate change adaptation strategies for biodiversity management assembled from 112 scholarly articles	2009	[18]
EBM Principles in the Western Pacific Context	2010	[65]
A more unifying framework for ecosystem-based sustainability: a Radical Ecosystem Approach	2010	[66]
Principles to guide climate change adaptation	2010	[67]
Challenges and solutions for protected area management in Ukrainian Transcarpathia	2011	[68]
Strategies of conservation planning and management in the face of climate change	2011	[69]
Vulnerability index for protected areas and consequent options of action	2013	[33]
Generic options of action for integrative conservation	2013	[17]
Key characteristics of climate-smart conservation	2013, 2014	[70], [71]
Guidebook for risk-robust, adaptive and ecosystem-based conservation of biodiversity (MARISCO)	2014	[72]
Strategic options to adapt to climate change extracted from literature	2015	[29]
Lessons learnt from case studies applying Adaptive Management of vulnerability and RISk at COnservation sites (MARISCO)	2015	[73]

**Table 3:** Principles and criteria of climate change-robustness (comprehensive list of principles and criteria with full titles, further description, rationale and references can be found in S1 Table).

Principles	Criteria	Rationale
1 Addressing climate change	1.1 Climate change in situation analysis	If conservation management is to be effective under climate change, this must be actively addressed in planning and be adopted as an active and constitutive factor of the system(s) to be managed.
	1.2 Climate change in goal setting	
	1.3 Climate change in strategies	
	1.4 Climate change in monitoring and research	
2 Ecosystem functionality & resilience	2.1 Prioritize higher-order systems	Ecosystems change but they change even more and faster under climate change. Therefore, they need to be as functional as possible to support their properties of self-organization and self-regulation. Ecosystem functionality is thus important for the maintenance of ecosystem resilience and adaptive capacity, which are all essential for facing and dealing with climate change.
	2.2 Prioritize functionality over patterns	
	2.3 Flexible protection	
	2.4 Biomass diversity and network	
3 Adequate spatial dimension	3.1 Functional ecological boundaries	Climate change has many impacts biodiversity, some of which occur with large spatial dimensions such as species’ and systems’ spatial shifts. It is therefore necessary to consider influencing factors and surrounding regions on a broad scale and to increase the functionality of conservation targets (ecosystems) in order to buffer those changes and to account for them. Applying adequate spatial dimensions is therefore essential for effective conservation planning and management under climate change.
	3.2 Continuity and connectedness	
	3.3 Regional context	
	3.4 Adjacent ecosystems	
4 Adequate time dimension	4.1 Long-term perspective	Most (climate and climate-induced) changes occur over long time periods and need to be addressed early enough but with a far time horizon to ensure success of conservation measures.
	4.2 Future changes	
	4.3 Activities with different time horizons	
	4.4 Long-term impact of activities	
5 Holistic knowledge management	5.1 Knowledge tracking	Climate change not only affects biodiversity but also other systems, such as systems of human land use, which might ultimately affect biodiversity. Further, climate change increases the complexity of conservation and other systems and of their interaction; it generates higher rates of uncertainty. For addressing complexity and uncertainty, a holistic management of knowledge and non-knowledge is necessary. In order to manage a system effectively it is important to know as much about the system as well as about climate change impacts as possible and to use different sources of knowledge. In order to deal with uncertainty it is equally important to keep track of non-knowledge.
	5.2 Diverse knowledge forms
	5.3 Diverse disciplines
	5.4 Knowledge exchange
6 Systemic and strategic coherence	6.1 System interaction	Climate change does not only affect a single system but also its subsystems and the superior system, even with different kinds of impact. Those changes of nested or larger systems may then indirectly also affect the system in focus. Hence all system levels need to be considered and their management needs to be aligned.
	6.2 Vertical nestedness	
	6.3 Horizontal coherence	
	6.4 Inter-protected area management	
7 Adaptive management	7.1 Iterative planning	Climate change is connected to a high degree of uncertainty and non-knowledge. Due to its iterative and error-friendly character and strong focus on monitoring and feed-back mechanisms adaptive management allows for managing under uncertainty. With adaptive management approaches (climatic) changes can be discovered and integrated into planning early. It allows for in-time adaptation of goals, targets, strategies and actions to increase conservation effectiveness.
	7.2 Systematic monitoring	
	7.3 Adaptive target and goal setting	
	7.4 Evaluation of effectiveness	
8 Proactive risk management	8.1 Precautionary principle	Climate change comes with great uncertainties and increases the risk potential for conservation systems, for example due to increased extreme events and higher weather variability. Climate change does not only affect conservation target per se but also other systems such as land use systems, which might increase the risk for conservation systems. Proactive risk management acknowledges that anticipatory rather than reactive approaches to conservation are essential when dealing with climate change. It facilitates the preparation for potential changes through anticipation and risk analysis. This enables adapting strategies before changes really affect a system, not only afterwards, and can save costs and ensure effectiveness.
	8.2 Future target vulnerability	
	8.3 Scenario planning	
	8.4 Robust strategies	
9 Institutional capacity building	9.1 Decentralization and responsibility	Only with sufficient (institutional) capacity, especially to deal with climate change but also in general, effective management in the face of climate change is possible.
	9.2 Transdisciplinarity of team	
	9.3 Knowledge and research capacities	
	9.4 Methodological training	
10 Public accountability and acceptance	10.1 Participation	Climate change poses a particular challenge to conservation managing systems such as protected areas that in many cases tend to have low management effectiveness even without climate change. In order to successfully deal with aspects of climate change in management it is necessary to guarantee a basic functioning of the conservation management system. The acceptance and the support of the public represent preconditions for effectiveness. Resistance, conflicts and counteraction minimizing opportunities to deal with climate change will hamper management. Further, any effort towards climate change adaptation will be ineffective without public support. Protected areas do not function in isolation but within a local and/or regional system. Therefore, conservation under climate change requires an integrative approach that includes all people in and around protected areas, especially land users. They need to be considered an essential part of (conservation) systems.
	10.2 Regular public reporting	
	10.3 Acceptance-increasing strategies	
	10.4 Public information	
11 Matrix and stakeholder management	11.1 Regional context	Conservation systems are connected with and embedded in other systems such as human (land use or political) systems. Climate change is a global issue and therefore affects all those systems equally. Since most threats and influencing factors on conservation targets occur in the surroundings of conservation sites, those surroundings are important for the connection of individual sites. Therefore, it is essential to pursue an integrative ecosystem management approach to account for (climate) changes in all relevant systems and to support conservation effectiveness. In times of climate change, conservation management is facing the need to consequently and effectively implement strategies that exceed current dimensions and to engage in cooperation with land users and stakeholders much more. It is important to not only acknowledge but also communicate the higher relevance of conservation and climate change to society than traditionally considered.
	11.2 Stakeholder cooperation	
	11.3 Concerted strategies	
	11.4 Cooperative ecosystem-based climate management	

### Plan evaluation procedure

The analysis of management plans involved a team of four coders (the leading author and three research assistants), in order to reduce personal bias. The three research assistants were thoroughly introduced to the protocol and instructed in the manner of scoring. In order to ensure scoring consistency, the coders test-coded several trial-plans prior to the evaluation. Based on the tests the protocol was revised and adapted to make it more robust to scoring by different coders. The final protocol was then used to conduct the evaluation of the 60 management plans. Each coder coded a different set of plans individually first, followed by a process of review by the lead author and open discussion among evaluators until a consensus on the scores was reached.

The degree of accordance of the eleven principles in the management plans was evaluated by scoring the plans against each of the respective four criteria of each principle on a 0–2 scale similar to the analytical approaches of previous studies on plan quality (e.g., [33, 44, 46, 47, 74]). In the present study a score of 0 was given if the criterion was not at all met by the plan or 1 if the criterion was only implicitly acknowledged or partly reflected in the plan without thorough explanation. The highest possible score of 2 was given if the criterion had been fully and comprehensively addressed by the plan. For example, considering criterion 1.1 *Climate change in situation analysis*, a management plan would get a score of 0 if climate change was not mentioned at all in the situation analysis. The plan would receive a score of 1 if it mentioned climate change as a factor influencing conservation objects without further explanation, or if it elaborated on climate change for only a small fraction of the situation analysis while neglecting most parts of it. A score of 2 could be reached if climate change was comprehensively included, elaborating on the impact on biodiversity as well as other factors, such as land use or infrastructure development. The individual scores for each management plan are summarized in S3 Table.

### Data analysis

We analysed the data in three stages. First, we calculated the general climate change-robustness index for each management plan by summing the actual scores of each of the 44 criteria (max. score 88). This index represents the overall performance of the 60 plans against the criteria of the evaluation framework. For an easier assessment, we additionally standardised and normalized the original plan score in percent, and used descriptive statistics to assess overall performance. In the second stage, we tested for statistical significance for the contribution of individual site characteristics of the protected areas to climate change-robustness in “R” [75] using the package “ggplot2” applying a Pairwise Wilcoxon Rank Sum Test for protected area category and the Spearman’s Rank Correlation for area size in case of Natura 2000 sites. In the third stage we conducted an item performance analysis on the basis of techniques frequently used in plan quality evaluation studies [44, 47, 76, 77]. We assessed and compared the performance of the eleven principles and the 44 criteria across all management plans of the sample from three perspectives: their rate of accordance (item breadth score), the quality or degree of their accordance (item depth score) and their total performance (total item score). The three item scores were calculated as follows:

1. Item breadth score: number of plans that address the item/number of plans in sample (score 0–1)
2. Item depth score: total score of all plans that addressed an item/ number of plans that addressed the issue (standardised score 0–1)
3. Total item score: item breadth score + standardised item depth score

We used the breadth analysis to measure the extent to which each of the principles and criteria was addressed across all management plans, i.e. the proportion of plans that addressed the individual principle or criterion. The score was given on a 0–1 scale for all items. The depth score measures to what extent each individual principle and criterion was addressed by the plans, meaning the level or intensity of accordance for each principle. The depth score for principles reaches a value between 0 and 8 and was standardised to a 0–1 scale (where 0.125 is the lowest achievable value if at least one plan adopted the principle; the value 0 is assigned to principles that were not reflected at all in the plans). With regard to the criteria, the depth score results in a 0–2 scale that was standardised to a 0–1 scale (where the lowest achievable value is 0.5 if at least one plan adopted the criterion, and the value of 0 is reached if none of the plans adopted the criterion). The total item score combines the breadth score and depth score to describe the overall performance of the individual principles and criteria. Finally, we classified principles according to their rate and degree of adoption (i.e. their breadth and depth rates) as indicated in Table 4.

**Table 4:** Classification of performance of climate change-robustness principles as per rate and degree of accordance.

**Degree of adoption** (depth score)	**Rate of adoption** (breadth score)			
	**Very high** (>0,75)	**High** (0.5–0.75)	**Low** (0.25–0.5)	**Very low** (<0.25)
**Very high** (>0.75)	Very strong	Very strong	Strong	Moderate
**High** (0.5–0.75)	Very strong	Strong	Moderate	Weak
**Low** (0.3–0.5)	Strong	Moderate	Weak	Very weak
**Very low** (<0.3)	Moderate	Weak	Very weak	Very weak

## Results

### Overall climate change-robustness—Performance of management plans

**Fig 1:**
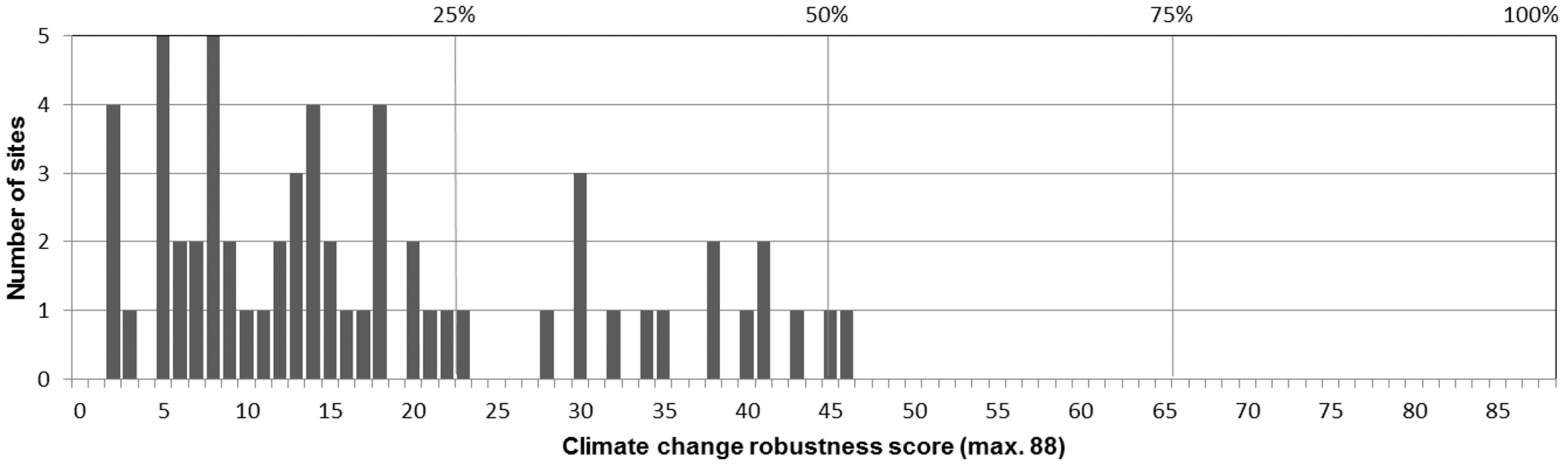
Distribution of climate change-robustness scores for the 60 protected areas analyzed. The climate change-robustness is the sum of the 44 individual scores for each management plan reached by scoring all plans against each of the respective four criteria of each principle on a 0–2 scale. Mean value = 18, median = 14.

**Fig 2:**
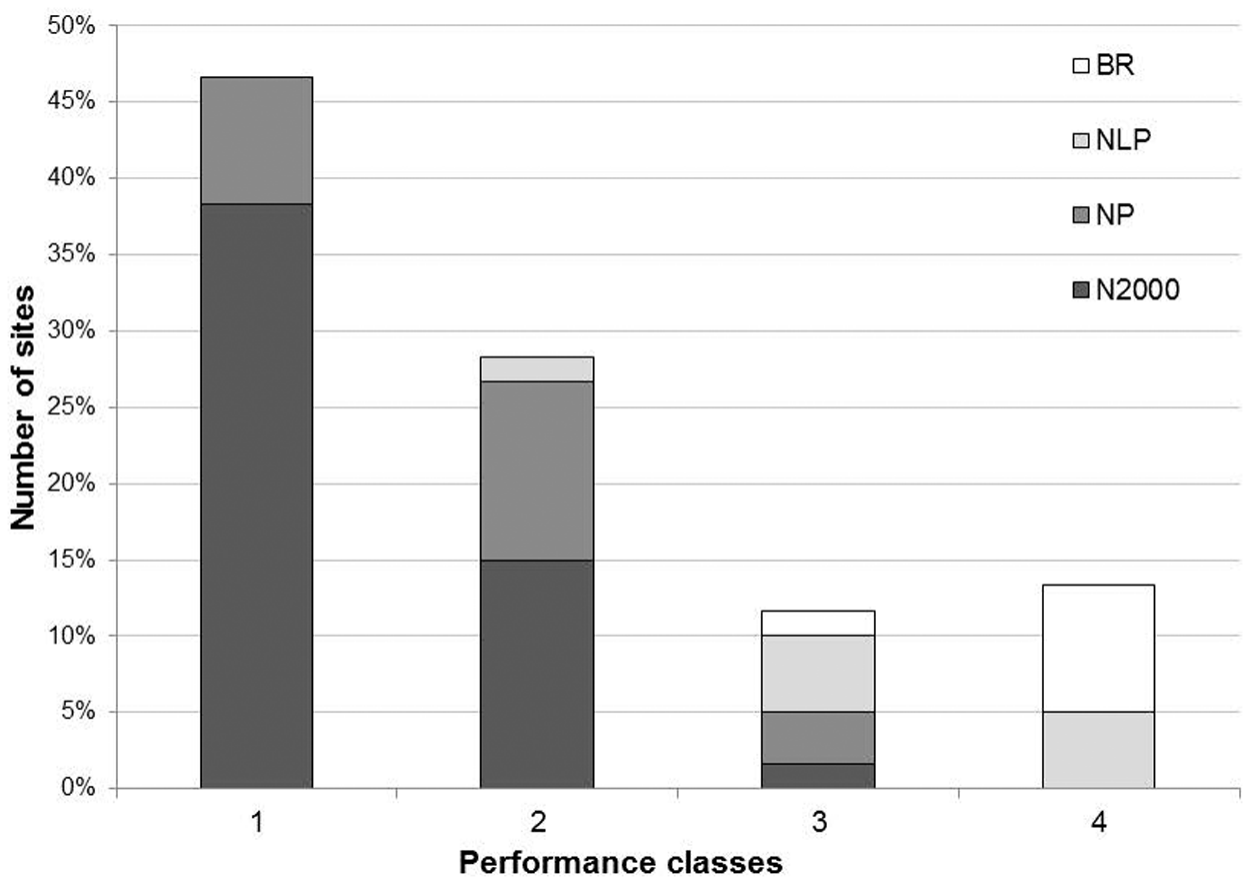
Distribution of protected area plans relative to the maximum performance of climate change-robustness. The performance of plans was normalized relative to the maximum performance of a score of 46 out of a maximum of 88.

The climate change-robustness values of the 60 sites ranged from 2 (2.3% of the maximum score; four sites) to 46 (52.3%; one site), with a median value of 14 and a mean value of 18 (20%; Fig 1). Two-thirds of the protected areas studied (n = 60) achieved a climate change-robustness index value equal to or lower than 20% of the maximum score. These 40 sites included one NLP, nine NP and 30 Natura 2000 sites; 30 of these management plans had been recently completed (2010–2013). 45 plans (75%) reached 50% and less of the score of the best-performing plan (Fig 2). Less than a third of the plans (16) achieved an index score of more than 25%, of which only two sites scored slightly more than 50% of climate change-robustness.

**Fig 3:**
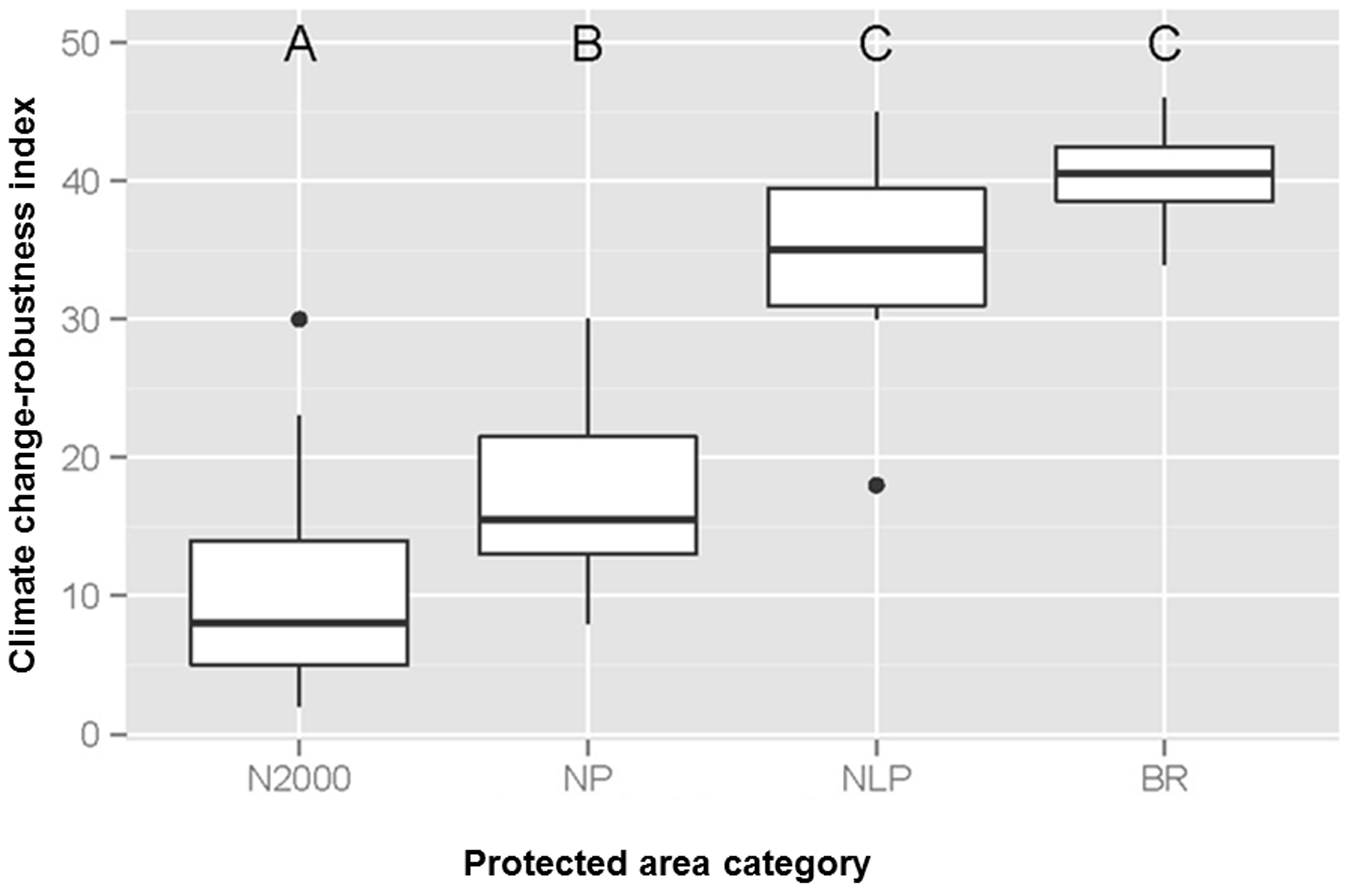
Relationship between the four protected area categories and climate change-robustness. BR = biosphere reserves, NLP = national parks, NP = nature parks, N2000 = Natura 2000 sites; different capital letters indicate significantly different groups.

**Fig 4:**
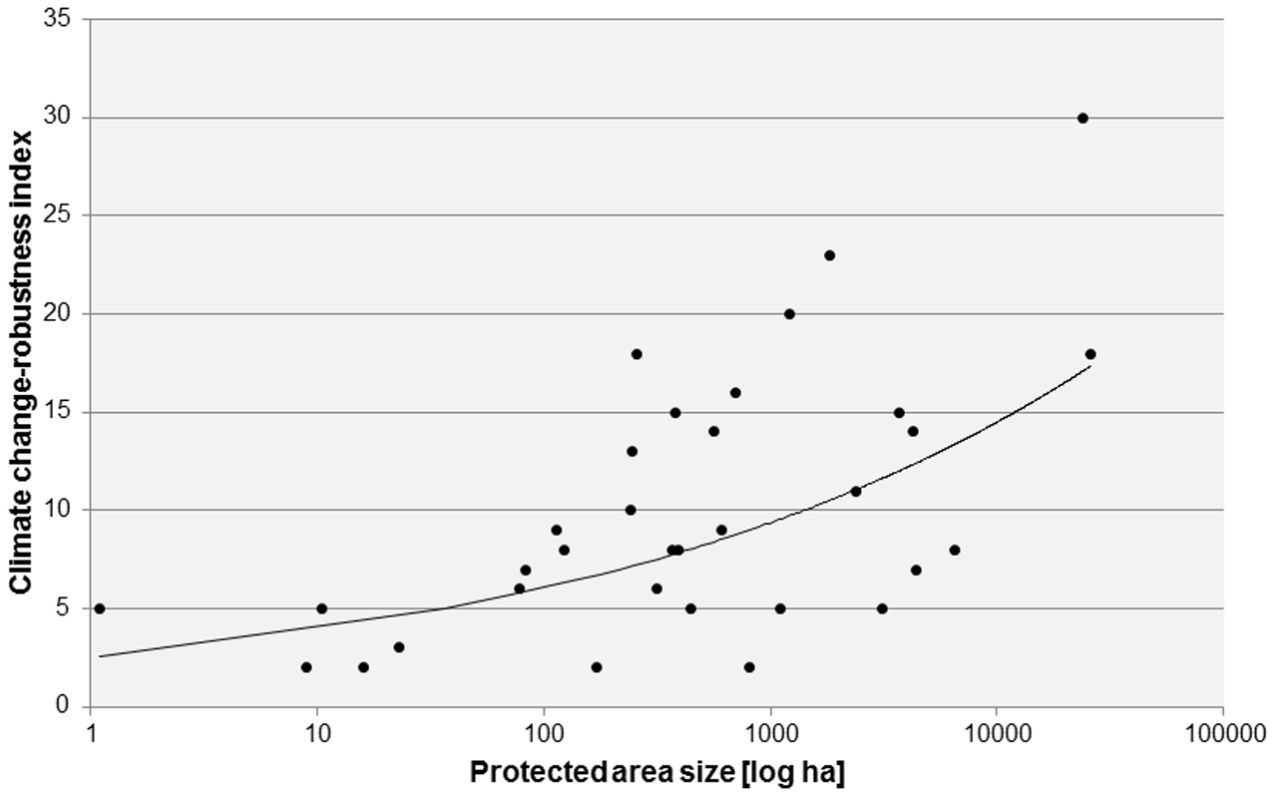
Relationship between protected area size and climate change-robustness of Natura 2000 sites. R = 0.58, p-value = 0.001406.

We found a strong relationship between the four protected area categories and climate change-robustness, where BR and NLP composed the most robust group with mean climate change-robustness values of 40 and 34 and median values of 41 and 35, respectively (Fig 3). Nature parks (mean = 18) and Natura 2000 sites (mean = 10) were significantly less robust. Natura 2000 sites were also the smallest protected areas where climate change robustness of their management planning significantly increased with area size (Fig 4).

### Performance of climate change-robustness principles

**Fig 5:**
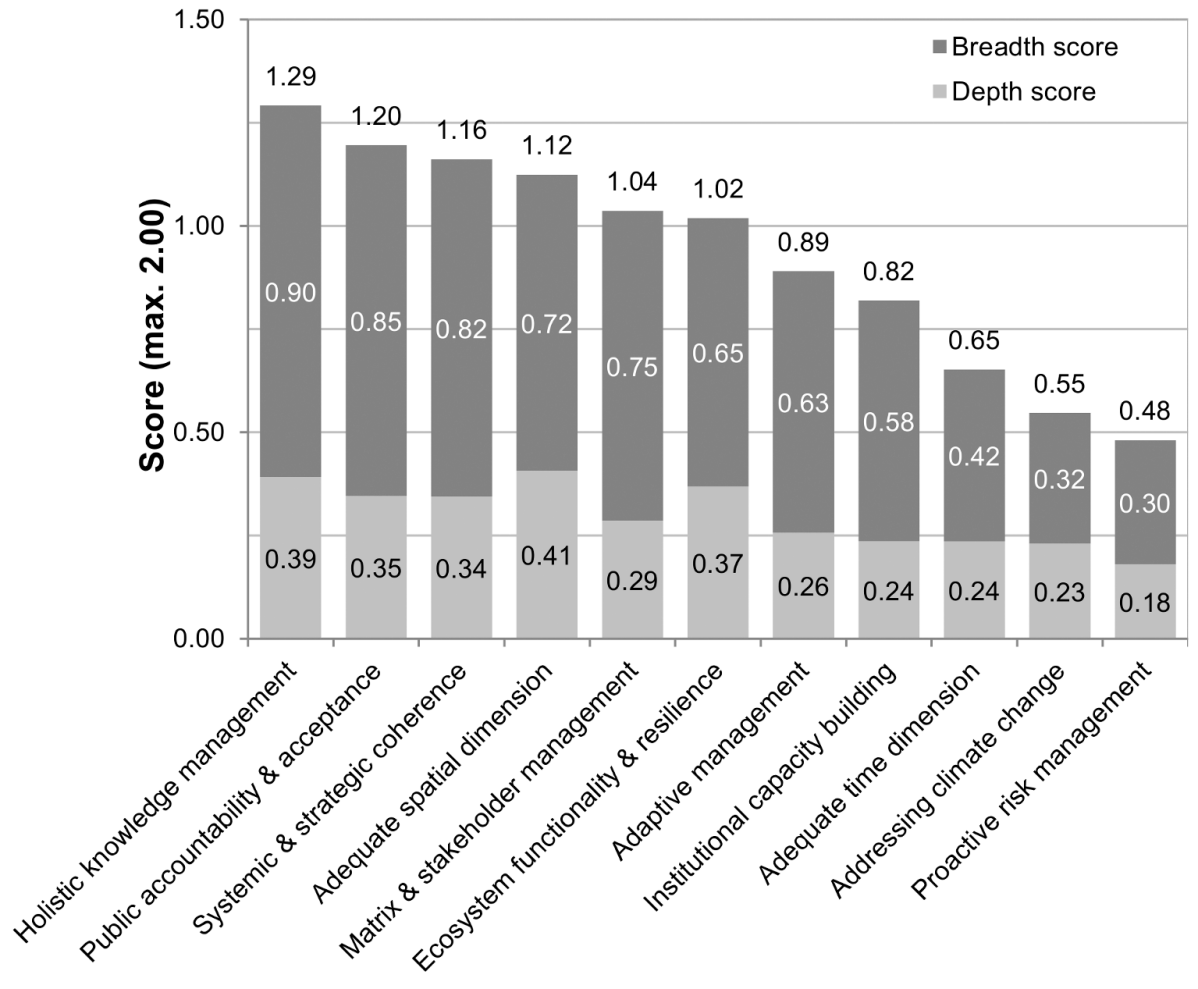
Standardised breadth, depth and total quality scores of the eleven robustness principles in descending order of total quality. Breath score = Number of plans that address the principle (max. 1.00), depth = intensity of addressing the principle (max 1.00), total quality = sum of breadth and depth score (max. 2.00).

**Fig 6:**
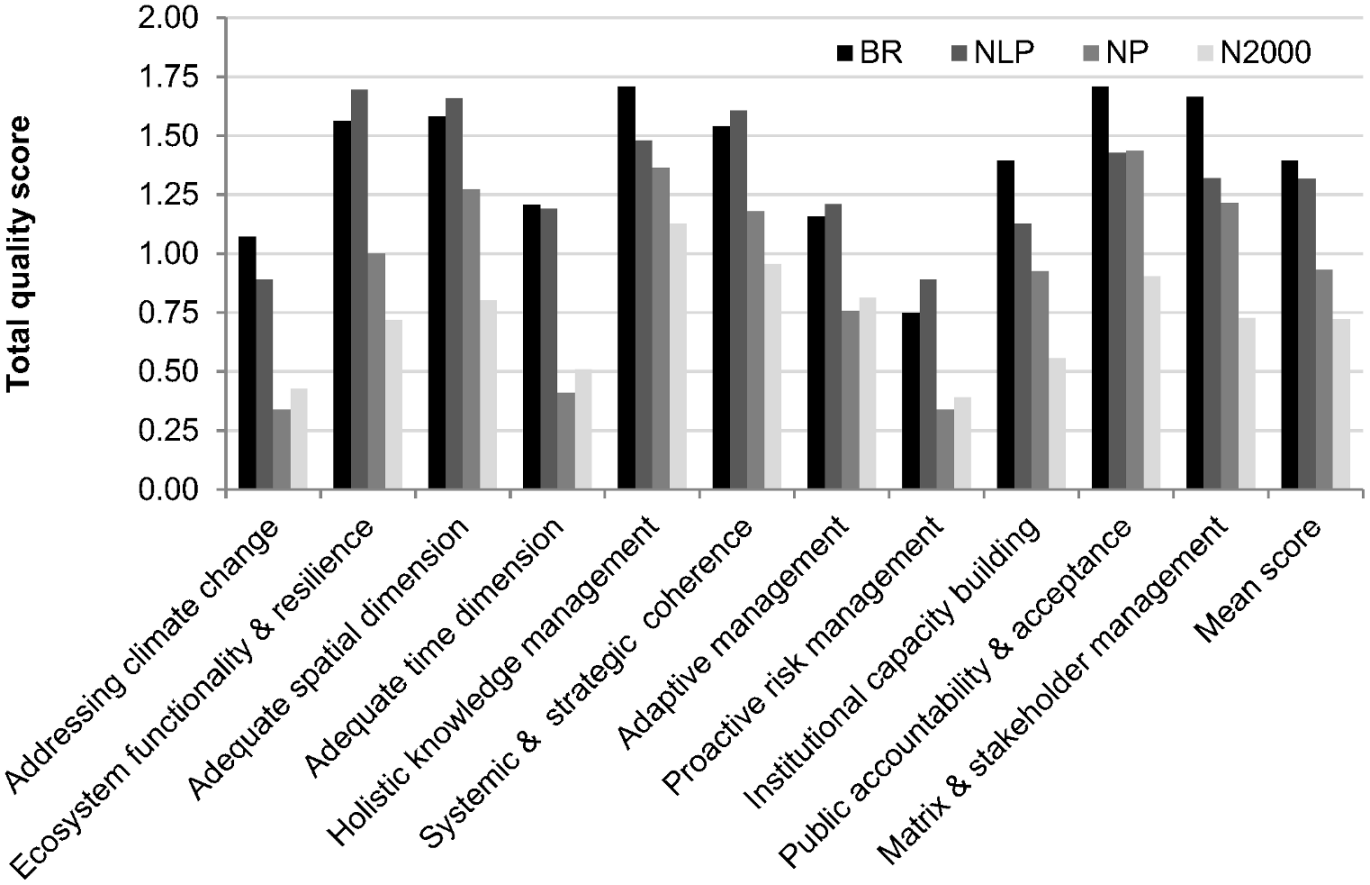
Total quality scores for the principles of climate change-robust conservation management sorted by protected area category. BR = biosphere reserves, NLP = national parks, NP = nature parks, N2000 = Natura 2000 sites.

All principles were reflected by the sampled management plans, but to differing rates and degrees (Fig 5). The principles were also applied differently in the four protected area categories (Fig 6). In order to structure the results, the principles can be classified into four groups depending on their rate (breadth score) and degree (depth score) of accordance: principles with strong accordance (3 principles), those with moderate accordance (3 principles), weak accordance (2 principles) and very weak accordance (3 principles) (compare Table 4).

#### Principles with strong accordance

Three principles had been strongly reflected by the analysed management plans: *holistic knowledge management (principle no. 5 as listed in Table 3 –P5)*, *public accountability and acceptance (P10)* as well as *systemic and strategic coherence (P6)*. With more than 80% coverage, their rate of accordance and hence acknowledgement was very high. However, their degree of accordance was rather low, with depth scores between 0.34 and 0.39, and very high scores were only reached in a few management plans. *Holistic knowledge management (P5)* attained the highest breadth score of all principles (90%). All of its four criteria scored relatively equally; the *integration of diverse knowledge forms (5*.*2)* prevailed in terms of coverage (breadth 70%) and the consideration of *different sectors and disciplines (5*.*3)* was most intensely reflected (depth 0.66). The majority of management plans were developed with a more or less intense *participation of the public (P10)*, or at least an acknowledgement of that principle (73%). In contrast, *informing the public* on climate change *(10*.*4)* and associated topics were almost hardly addressed (10% coverage). A similar minor heterogeneity between criteria showed the principle of *systemic and strategic coherence (P6)* where *inter-protected area management (6*.*4)* was only weakly demonstrated (breadth 20%, depth 0.5) compared to the other three criteria.

#### Principles with moderate accordance

A moderate accordance could be identified for managing with *adequate spatial dimension (P3)*, *matrix and stakeholder management (P11)* as well as for managing for *ecosystem functionality and resilience (P2)*. The principle related to the *spatial dimension (P3)* of planning and management attained the highest degree of accordance (0.41) of all the principles. Two thirds of the plans approached a *spatially continuous management scope and connection to adjacent sites (3*.*2)*, but only around one third of the plans regarded conservation targets truly within a (n) *eco-regional context (3*.*3)*. *Matrix and stakeholder management (P11)* showed the second strongest heterogeneity between criteria after *institutional capacity building (P9)*. A high proportion of management plans considered the *regional setting of the protected area beyond the borders of the protected area* (*11*.*1*; 48%) and the associated *stakeholder cooperation* (*11*.*2*; 68%) with comparably high intensity (depth 0.66 and 0.67). However, the *adaptation and mitigation activities of surrounding land users (11*.*3)* and their engagement in *cooperative ecosystem-based climate management (11*.*4)* were not considered in more than 90% of the analyzed management plans. *Ecosystem functionality and resilience (P2)* was represented most by the management for *high levels of biomass*, *diversity and network (2*.*4)*.

#### Principles with weak accordance

Two principles reached a weak application status: *adaptive management (P7)* and *institutional capacity building (P9)*. The latter showed the highest heterogeneity between criteria of all principles, with two comparably well-reflected criteria (*decentralization and clarification of responsibilities (9*.*1)*, *transdisciplinary planning team (9*.*2))*, and two criteria concerning climate change capacity building *(9*.*3* and *9*.*4)* not addressed at all. The principle of *adaptive management (P7)* was mainly implemented in terms of a *systematic monitoring system (7*.*2)*. An *iterative planning approach (7*.*1)* with adaptive target and goal setting was seldom attempted in management plans sampled (22% and 20% respectively, depth 0.5).

#### Principles with very weak accordance

Three principles were very weakly met by the management plans: *adequate time dimension (P4)*, *addressing climate change (P1)* and *proactive risk management (P8)*. The consideration of an *adequate time dimension (P4)* found their way into planning mainly by adopting a *long-term perspective (4*.*1)* for planning and management and in *activities with different time horizons (4*.*3)*. In contrast, *future changes of conservation targets (4*.*2)* and the *long-term impact of activities (4*.*4)* were hardly considered in strategy development. Climate change was mainly *addressed in situation analyses (1*.*1)*, but much less considered *in goal setting (1*.*2)*, *strategy design (1*.*3)* as well as in *monitoring and research (1*.*4)*. *Proactive risk management (P8)* was the principle least often (BS 0.3) and least intensely (DS 0.18) considered in management planning. Here, slightly more attention was given to the *precautionary principle (8*.*1)* as well as *future analysis of risks and vulnerabilities (8*.*2)* of conservation targets than the other two criteria.

#### Performance of principles and protected area category

BR, NLP and NP generally acknowledged the principles of climate change-robustness with accordance rates of more than 50% for most principles. BR and NLP had the most diverse competences in terms of meeting the principles. BR and NP performed best in terms of *holistic knowledge management (P5)* and *public accountability and acceptance (P10)*. They appeared especially strong in terms of *strategies to increase the acceptance (10*.*3)* for the site and *stakeholder cooperation (11*.*2)*. The main strength of NLP was to manage for *ecosystem functionality and resilience (P2)*, particularly *prioritizing higher-order systems* (2.1) and *functionality over patterns* (2.2). One further competence was managing and planning with *adequate spatial dimensions (P3*), particularly considering and managing *adjacent ecosystems/regions as areas of influence (3*.*4)*. Natura 2000 sites attained the highest total quality scores for *holistic knowledge management (P5)* as well as *systemic and strategic coherence (P6)*. However, their scores were still distinctly lower than those of the other protected area categories.

#### Performance of individual criteria

We found the most frequently reflected criteria (and the ones with the highest total scores, compare S4 Table) were connected to stakeholders and their incorporation in management (*10*.*1 public participation in management planning*, met by 73% of the plans; *11*.*2 stakeholder incorporation in management strategies*, 68%; *5*.*2 considering diverse knowledge forms*, 70%) and the strategic alignment of different planning levels (*6*.*2 vertical nestedness*, 65%).

The most intensively reflected criteria included very different fields of action: *strategies to increase and ensure the acceptance of the protected areas by the public* (*10.3*; depth 0.75), managing for *biomass*, *diversity and network (2*.*4)* within the protected area to maintain and enhance ecological functionality and resilience (depth 0.69), *managing beyond the borders of the protected area (11*.*1)* into the matrix (depth 0.67) as well as managing a protected area with a *continuous management scope and connecting to nearby ecosystems* (*3*.*2*, depth 0.67). The first three criteria were only addressed by a minority of plans (45–48%) but the latter one was considered by 60% of the management plans.

Especially those criteria associated with dealing with an uncertain future could be found in very few plans (5% and less) and with very low intensity (depth 0.5, on a 0.5–1 scale). Furthermore, the extension of strategy design to include *future changes of conservation targets (4*.*2)*, *scenario planning (8*.*3)* and identifying and prioritizing *robust or non-regret strategies (8*.*4*.) could only be found in five plans (8%). Institutional capacity building with regard to *climate change research and management methodologies (9*.*3*, *9*.*4)* was not treated at all in any of the analyzed plans.

## Discussion

### Conservation management planning and climate change-robustness

One purpose of our study was to assess climate change-robustness of protected area management plans in Germany. Our findings suggest the climate change-robustness of management plans of German protected areas is rather low. The majority of plans scored below 25% of the maximum score, lacked concreteness and neglected the issue of climate change adaptation. Our results underpin those of Kreft et al. [33]. According to their climate change vulnerability index applied to German protected areas, most of the 121 assessed protected areas in Germany can be categorised as highly vulnerable against climate change; none of the sites showed low vulnerability [33]. The only two sites we found scoring above 50% of the possible score showed great differences in their individual site characteristics. One area was a NLP in Bavaria with a prevalence of forests and mountainous terrain; the plan was accomplished by the park administration in 2010. The other area was a BR in Mecklenburg-Western Pomerania with an open landscape in the northern plains and a management plan prepared by an external contractor in 2003. Our results indicate that it is not individual site characteristics per se that affect climate change-robustness, but primarily the protected area category. BR and NLP seem to be most robust, while NP and Natura 2000 sites are particularly deficient concerning climate change. Reasons could include the differences in guidelines, requirements and the administration of different protected area categories, but also differently structured competences and resources available.

The higher robustness performance of BR and NLP is not surprising, since their definition and legislation inherently provide much better for climate change-robustness than do NP and Natura 2000 sites. The underlying concepts of BR and NLP foster greater motivation to meet principles of the Ecosystem Approach. However, the relatively high robustness scores among BR and NLP were still rather low in absolute terms. This should give cause for concern since a large part of the principles and their criteria can be considered best practice conservation, as they have already been adopted in other concepts such as the Ecosystem Approach. Even after one and a half decades, the Ecosystem Approach still appears to remain ‘stuck in the clouds’ [15]. Similarly, NP per definition–as large landscape-scale protected areas–also have an appropriate fundament theoretically favouring climate change-robust management, but many of them exhibit weaker enforcement, administration and implementation of that definition.

Our results underline the deficiencies of the Natura 2000 complex regarding climate change (adaptation) that have been previously identified and discussed elsewhere [78–80]. One important problem is the static conceptualisation of Natura 2000 protection goals, which require a fixed list of annex species and habitats to be conserved on-site [78, 80]. In addition, Natura 2000 implementation follows country-specific idiosyncrasies. In the case of Germany, the sheer number of over 5,000 Natura 2000 sites makes it a tantalising effort for the conservation administrations to provide every site with a management plan, even if it was a simple one. The quality of the plans thus has become second priority. The guidelines for Natura 2000 management planning generally do not develop instructions for climate change robustness, which is another factor. For instance, the newest version of the handbook for management planning of sites protected under the Habitats Directive of the EU in the German federal state of Brandenburg does not refer to climate change at all [81]. The previous version covered this topic in several contexts, although only superficially. It must be concluded that innovations towards climate change-robustness have not only been lacking but even regressing. This is also reflected by our results: most of the younger plans in our sample belong to Natura 2000 sites, which attained lower robustness scores relative to large, national protected areas. This stands in contrast to the intuitive assumption that knowledge increases over time and is in turn incorporated into management guidelines [48, 82]. It is noteworthy that the most vulnerable conservation sites in Germany (i.e. the Natura 2000 sites as also identified by Kreft et al. [33]) have the least climate change-robust conservation management planning. Moreover, as we have indicated, among the Natura 2000 sites management planning of the smaller areas tends to be less climate change-robust than that of larger Natura 2000 sites. This seems quite maladaptive as smaller areas are inherently more vulnerable to climate change and other threats, and thus are areas that would require a more robust management planning.

The protected area management plans evaluated in our study differed enormously in their quality and contents (and assumingly in their function in actual management). Nolte et al. [37] found management planning to be one of the weak elements with regard to protected area management effectiveness. The present study does not account for unofficial or non-published internal management documents which might in fact play an important role with regard to climate change adaptation efforts [29, 74]. For instance, the management framework *(Rahmenkonzept)* of Schaalsee Biosphere Reserve was published in 2003 [83] and remains the official guiding document. Climate change is only scarcely considered in this document. Since 2007 the BR has engaged very actively in climate change mitigation and adaptation with several projects, cooperative partnerships and studies (e.g., [84]) which are mainly presented on the website of the BR [85]. At the same time, it is questionable in how far the contents of even well-composed management plans find actual implementation. Judging from our experience, we would argue that an evaluation of actual management performance and effectiveness might produce even lower climate change-robustness scores. In many cases, in Germany, Natura 2000 sites have assigned neither staff exclusively in charge nor regular resources for implementation. Often the areas are very small and do not have appropriate buffer zones. Due to the lack of demarcation they are often not even recognizable for visitors or land users. There is a clear risk that management plans might not actually be used as the basic and guiding instruments for management. The low values of climate change-robustness might represent the tip of an iceberg of management deficiencies. If management plans constitute a key element of adaptation to climate change, their quality, functional role, preparation and implementation need to be improved.

We found the principles of climate change-robustness were reflected very differently in the plans. This confirms the notion that standards in conservation planning are lacking–which was also concluded from a study on German county-level conservation plans (*Landschaftsrahmenpläne*, regulating conservation on the county level) [48]. A similar finding applies to many climate change adaptation plans beyond nature conservation [46, 47, 74]. Therefore, existing adaptation guidance is used inconsistently. This does not preclude quality plans, but it certainly increases the risk of failure [74].

### Principles of climate change-robustness

#### Achievements

The strongest accordance with the principles of climate change-robust conservation management was achieved in terms of *holistic knowledge management (P5)*, *public accountability & acceptance (P10)* as well as *systemic & strategic coherence (P6)*. We presume that the strength of principles with higher performance partially arises from other discourses and discussions in connection with conservation than that of climate change adaptation. Public participation and acceptance as well as the connectivity of ecosystems are topics that find attention in many other contexts. Nonetheless, they support climate change-robustness of protected areas and should even be more promoted in the light of adapting to climatic or any kind of change. The high performance of the principle of *holistic knowledge management (P5)* is possibly grounded on the high motivation of administrations in Germany to compile and document knowledge about protected areas, which for many of them–in our experience–is appreciated as the most important planning outcome. Gathering, analysing and discussing knowledge indeed generates new understanding and awareness. However, translation into practical management, i.e. implementation, might turn out to be deficient, making knowledge accumulation of little use. This knowing-doing gap is a common problem in conservation [86, 87].

#### Deficiencies

The most serious deficiencies we encountered were associated with principles dealing with the future and uncertainty in particular: planning with *adequate time dimension (P4)*, *addressing climate change (P1)* and *proactive risk management (P8*). This corresponds with the most prominent climate change adaptation deficiencies identified in other studies and contexts [29, 48, 74]. Additionally we concluded that NP showed the greatest deficiencies in terms of those three principles. Management units in charge of Natura 2000 sites exhibited very low degrees of accordance for all of the principles (most depth scores below 0.25) with very different rates of acknowledgement of their importance.

The encountered deficiencies appear to be tightly connected with the total lack of capacity building strategies for protected area staff with regard to research abilities and methodological training for climate change adaptation across all evaluated plans. The wish and perceived need to increase the knowledge base before acting prevails. This issue was also encountered in 57 climate change adaptation plans in developed countries [74]. Because of uncertainty and the lack of a culture dealing with non-knowledge and mistakes, managers seem to feel not strong enough and legitimated to ground management decisions on projections or scenarios. In conversations (in Brandenburg) they often insist in the judgement that current threats to biodiversity are more pressing than future changes. They seem to be more comfortable with reactive management, which is easier to justify, even when it is clearly coming too late and likely to fail. From many discussions we know that the reason could be even more trivial, but still alarming: Managers are simply overwhelmed by day-to-day management and have no resources for thinking about a more complex and future-oriented management. However, this greatly inhibits climate change adaptation. Also Geyer et al. [29] identified a serious lack of confidence in proactive and future-oriented thinking. Other obstacles associated with the most deficient principles identified in other studies include a lack of resources and manpower, established management habits that conflict with a systematic learning process, static and incoherent legislation as well as restrictive policies and terms of reference for certain protected areas [27, 29]. Not questioning the changeability of such barriers (however difficult they might appear to overcome) may itself be considered an obstacle to the adaptation process [88].

### Recommendations for practical applications

#### Improving conservation management planning

Undoubtedly, there is the need for better management plans in conservation [37]. Besides the quality of plans and their applicability, the process of planning also needs to be improved. It is inevitable to make conservation planning more systematic, strategic, adaptive, iterative and target-oriented. The *Open Standards for the Practice of Conservation* are in use at many conservation sites all over the world and have proven a valuable methodology for strategic adaptive conservation planning. This methodology makes management leaner, easier, more transparent and participatory in terms of both design and implementation [89]. MARISCO, an approach derived from the *Open Standards*, facilitates ecosystem-based adaptation to climate change and embraces risk management. It has been tested in numerous conservation projects all over the world [72, 73, 90]. Such management planning instruments address many shortcomings of current conservation management and take up on the presented principles of climate change-robustness. They are applicable on all planning levels regardless their size and organisational structure and represent a promising tool to bridge current planning and communication gaps in conservation planning [91, 92]. Their standard terminologies typically ensure high comparability between planning levels and different sites and can thus facilitate better coherence in target setting as well as in aligning strategies across different institutional levels [91]. Experience has also shown that those approaches are very useful to foster mutual learning. Further, guidelines on management planning, the participation in quality programmes (such as the German nature park quality campaign [51]) and international networks (like the Natura 2000 network or the UNESCO MAB Programme) as well as effectiveness evaluation programmes exert an additional influence on the quality of protected area management and its climate change-robustness. Protected areas would be well advised to comply with such guidelines and programmes and recognise them as chances to make management more (climate change-) robust.

#### Adopting principles of climate change-robustness in conservation management

If protected areas shall maintain their functions such as conserving biodiversity in times of (climate) change, their management will have to become more robust against such change. Our evaluation framework provides eleven principles and 44 criteria for climate change-robust conservation management. These principles and criteria are designed to serve as guidance on different planning and management levels. Protected area managers may use these principles in their planning activities, their daily work, for identifying area-specific shortcomings and strengths and thus enhance robustness of the site they are responsible for. Conservation policy might equally be guided by these principles. Drawing on principles and criteria identified as most deficient we consider it acutely necessary

- to consider future changes in general and climate change in particular in all steps of conservation management planning,
- to enhance cooperation between protected areas and to align their management,
- to take particular responsibility for ecosystem-based adaptation and climate management in protected areas and also their surroundings involving all relevant stakeholders,
- to build strong and systematic monitoring systems to measure goal achievement and management effectiveness and inform management planning,
- to build capacity for climate change management and install persons or larger management units that are commissioned to particularly deal with climate change and with climate change mitigation and adaptation,
- to accomplish with the information and education mission of protected areas in terms of climate change and its implications on local, regional, national and international level, and thus
- to inform and educate about options of mitigation and adaptation to climate change, ecosystem-based adaptation and climate management.

In addition, or as an alternative to new climate change-specific guidelines, existing guidelines for protected area management should be revised with regard to climate change adaption. Specific checklists for climate change-robust management need to be compiled, using, for example, the principles presented in this study or similar guidelines elaborated for conservation in Brandenburg, Germany [93, 94]. Sites protected within the Natura 2000 complex in particular would benefit greatly from such guidelines. This would not only increase climate change-robustness, but also improve management planning in general, which in many cases is currently suffering from substantial deficiencies. Surely, the process of climate change adaptation needs to be tailored to the site and to local needs [27, 74]. Nevertheless, general guidelines on climate change adaptation and specific principles for climate change-robust management can provide a framework into which protected area managers can fit their site-specific planning and actions.

#### Identifying and overcoming barriers for climate change adaptation

An analysis of obstacles to a successful adaptation of conservation management to climate change is essential. Specific frameworks for diagnosing barriers are available (e.g., [88]). Many perceived limits in fact turn out to be barriers that, once identified as such, can be overcome with pertinent effort. For example, the application of adaptive and risk management methodologies is often seen difficult in the frame of existing legal regulations. However, in many cases the reservation, insecurity and entrenched habits of planners and managers can be stronger impediments to climate change-robust management than those generated by existing legislation.

#### Fostering communication, collective learning and knowledge exchange

Collective experiential knowledge plays an important role in conservation [95] and might be particularly useful for climate change adaptation. It has been suggested that the establishment of open-access databases of management plans might facilitate collaborative learning, knowledge exchange and communication between conservation actors [33, 91]. Such exchange could include protected areas managers and other conservation actors as well as experts and researchers. It might benefit evaluation and revision of management plans, for example with regard to the principles of climate change-robustness. Different protected areas might have different competences regarding those principles. Thus, an exchange of knowledge and experiences might be useful to increase the intensity of their adoption. This holds especially for those principles that are already widely acknowledged and mostly lack adequate depth for satisfactory performance. Such cooperation might also make the identification of common barriers easier and facilitate the joint development of overarching solutions.

#### Linking planning, implementation and evaluation

The linkage between planning, implementation, evaluation and learning is the essence of adaptive management. It should be consolidated much more [37], both by practitioners on site level as well as on other levels of conservation management. The evaluation of management effectiveness needs to be understood as a learning process. It may improve management by facilitating individual and institutional learning as well as the exchange between conservation managers and other actors, by lending credibility to all actors of protected area governance, by raising awareness of importance of protected areas and also by contributing to reporting obligations [37, 96]. Moreover, protected area management effectiveness evaluations should account for climate change-robustness more explicitly, as the IUCN Green List of Protected and Conserved Areas Standard already demonstrates [97]. One may envision a kind of certificate for climate change-robust conservation management.

### Study limitations and future research opportunities

The most critical limitation of our study was that only the content of management plans, but not their actual implementation, was evaluated. However, addressing implementation would have gone beyond the scope of this study, or would have limited the sample of scrutinised protected areas to a low number.

Future research might apply different weights on individual principles and criteria according to their perceived influence and importance. Our study might provide some indication which principles could get assigned more weight in correspondence to their primary and immediate importance (e.g. addressing climate change, risk management, adaptive management, time dimension, managing for ecosystem functionality and resilience) and which ones might have less direct importance for climate change-robustness (e.g. individual criteria in terms of participation and acceptance, matrix management). In this case the overall climate change-robustness scores would probably even be much lower. Disadvantages of weighting are at least twofold. First, direct comparison of the performance of principles becomes more difficult. Second, unequal weighting means a loss in transparency of the principles and criteria framework.

It would be fruitful to extend and diversify the study sample for a more thorough assessment of climate change-robustness of conservation management. Thus, protected areas with less accessible management plans or different protection status could be included. In addition, the focus could be widened beyond protected areas and include plans and policies of other means of conservation management on varying levels (e.g., county-level plans, national biodiversity strategies). Finally, a comparison of protected areas in other countries with those in Germany is without doubt essential to reach a good understanding of the existing range of adaptation approaches to climate change.

In order to make conservation management more climate change-robust it would be beneficial to further explore factors that might explain variation in robustness. A closer link to local planning settings and processes might give valuable insight into causes for success and failures of climate change adaptation in conservation. Apart from basic resource capacities of protected area administrations, more delicate factors such as climate change risk perception of conservation managers, their planning capacities as well as personal values and beliefs can also play a role [29]. Details of the planning process (e.g., degree of public participation, consultation of external experts, available resources, responsibilities and mandates) as well as the governance context can also strongly influence planning, the quality of the plan and its implementation.

## Conclusion

Our study gives valuable insight into current deficiencies of accounting for climate change in protected area management planning. Although climate change adaptation in conservation has long been a focus of discussions and studies, this study sets a signal that its practical application to date has been slow and has not been conducted with enough rigor. By suggesting eleven principles with according criteria, we hope it may spark a discussion on (unused) options for making protected areas more climate change-robust. A good starting point is provided for further development towards climate change-robust protected area management since most principles were reflected by most of the plans suggesting that they are generally acknowledged as important. The lack of appropriate depth in addressing the principles of climate change-robust conservation management might imply that the necessary competence are still deficient in many protected area administrations and need to be developed. Existing and evolving competences and approaches might yield fruitful guidance for adapting to climate change.

## Supporting information

S1 Text Description of the four protected area categories sampled in the study: Biosphere reserves, national parks, nature parks and Natura 2000 sites. (PDF)

S1 Table Description and rationale of the principles and criteria of climate change-robust conservation management. (PDF)

S2 Table References of the 60 management plans analysed in the study on climate change-robustness of protected area management plans in Germany. (PDF)

S3 Table Evaluation scores of the 60 protected area management plans as assigned in the plan quality evaluation process. (PDF)

S4 Table Breadth, depth and total quality scores for the 11 principles and 44 criteria of the climate change-robustness index. (PDF)
