# Supplementary material for: Assessing climate change-robustness of protected area management plans—The case of Germany
Source: PLoS One. 2017 Oct 5;12(10):e0185972. doi: 10.1371/journal.pone.0185972 (PMC5628909; doi:10.1371/journal.pone.0185972)
Supplement: S1 Text — (PDF) [file pone.0185972.s001.pdf]

S2 Text. Description of the four protected area categories sampled in the study: Biosphere Reserves, National Parks, Nature Parks and Natura 2000 sites

According to the Federal Agency for Nature Conservation (BfN) "**National parks** (Nationalparke under Section 24 of the Act) are large-scale landscapes of national importance that are in – or are capable of evolving or being brought into – a state such that they show little or no human impact over most of their area. Nature should be allowed to take its course in them free of human exploitation or intervention. National parks help protect nature and biodiversity and provide safe havens for wild plants and animals." [1]
Most of Germany's national parks today are still in the development phase with continuing human impact. However, management of these parks set priority on dynamic natural processes for most territory in the near future [1, 2]. There are 16 NP in Germany, two of them only recently established, covering 0.6% of its territory.

Contrastingly, "**biosphere reserves** (Biosphärenreservate under Section 25 of the Act) are set up to protect large-scale natural and cultural landscapes. Their main aims are to preserve, develop or restore landscapes shaped by traditional diverse uses, along with their historically evolved diversity of species and habitats. They also serve as models for developing and testing sustainable operating methods in all sectors of the economy" [1]. In Germany, 15 of the 16 Biosphere reserves have been recognised by UNESCO under the Man and the Biosphere programme. They cover 3.7% of Germany's territory [1].

"**Nature parks** (Naturparke under Section 27 of the Act) are large-scale cultural landscapes in which protecting and maintaining habitat and species diversity are closely tied to their recreational function. They support sustainable tourism and sustainable use of the land." [1]. The 104 nature parks cover 27% of Germany's land surface. They have only recently been started to be acknowledged as protected areas/conservation sites – for a long time and in some regions they are still rather considered rural tourism development regions. Hence, German nature parks remain heterogeneous, especially regarding the achievement of the statutory aims of management and development. The Länder have different statutory requirements and choice of emphasis in nature park conceptual plans and charters. There are also differences in the general conditions provided by the responsible agencies and Länder. For example, only some Bundesländer require nature park plans. Also the administration differs between the Bundesländer. In some cases, nature park administrations are part of Länder environmental administrations; in others, the supporting organisation is a registered association (Verein) or a self-governing corporation set up by a group of local authorities (Zweckverband) [1, 3].

Natura 2000 is the largest coordinated network of protected areas in the world stretching across all 28 EU member states, over 18 % of the EU's land area and almost 6 % of its marine territory. The aim of the network is to ensure the long-term survival of Europe's most valuable and threatened species and habitats, listed under both the Birds Directive and the Habitats Directive across their natural range in Europe and to ensure that they are restored

to, or maintained at, a favourable conservation status. Natura 2000 is not a system of strict nature reserves where human activities are systematically excluded. Including strictly protected nature reserves, the approach to conservation and sustainable use of the Natura 2000 areas also centeres on people working with nature rather than against it. However, Member States must ensure that the sites are managed in a sustainable manner, both ecologically and economically. The management of Natura 2000 sites is therefore best done by working closely with the landowners and stakeholder groups in or around individual Natura 2000 sites in order to agree on the most appropriate ways to conserve the species and habitats whilst respecting the local socio-economic and cultural context [4]. The Habitats Directive demands that Member States

- Take appropriate conservation measures to maintain and restore the habitats and species for which the site has been designated to a favourable conservation status;
- Avoid damaging activities that could significantly disturb these species or deteriorate the habitats of the protected species or habitat types
- Follow a specific procedure in planning when plan or project will likely have a significant effect on a Natura 2000 applying an appropriate assessment determine its implications for the site [4].

1. BfN (Bundesamt für Naturschutz). Protected areas. 2015 [updated 10.09.2015; cited 2015 09.12.2015]. Available from: https://www.bfn.de/0308_gebietsschutz+M52087573ab0.html.

2. Dannenbaum M, Hüls N. Wild and beautiful : national parks in Germany. Berlin: Europarc Deutschland; 2011. 70 p.

3. Forst R, Scherfose V. Entwicklung und Perspektiven deutscher Naturparke. Naturschutz und biologische Vielfalt. 2010;104:189-95.

4. European Commission. Natura 2000. 2017 [updated 27 April 2017; cited 2017 20 June]. Available from: http://ec.europa.eu/environment/nature/natura2000/index_en.htm.
