# Supplementary material for: Assessing climate change-robustness of protected area management plans—The case of Germany
Source: PLoS One. 2017 Oct 5;12(10):e0185972. doi: 10.1371/journal.pone.0185972 (PMC5628909; doi:10.1371/journal.pone.0185972)
Supplement: S1 Table — (PDF) [file pone.0185972.s002.pdf]

Table S1. Description and rationale of the principles and criteria of climate change-robust conservation management.

Principles and criteria short titles (as used in the main article)	Principles and criteria in full notation	Description	Rationale	References
1 Addressing climate change<br>1.1 Climate change in situation analysis<br>1.2 Climate change in goal setting<br>1.3 Climate change in strategies<br>1.4 Climate change in monitoring and research	Addressing climate change<br>1 Address climate change and its impacts in situation analysis.<br>2 Address climate change in goal setting.<br>3 Address (ecosystem-based) climate change (management) in strategy design and action planning.<br>4 Address climate change in monitoring and research (monitor changes of climatic factors, biological, ecological and physical aspects of (climate change) impacts and climate change impacts in combination with other threats).	Recognize the relevance of climate change and its impacts for conservation management and address it in all subjects and all processes of conservation management (research, situation analysis, strategy development and monitoring activities).	If conservation management is to be effective under climate change, this must be actively addressed in planning and be adopted as an active and constitutive factor of the system(s) to be managed.	Baron et al. (2009)<br>Heller and Zavaleta (2009)<br>Mawdsley et al. (2009)<br>Freudenberger and Ibisch (2013)
2 Ecosystem functionality & resilience*<br>2.1 Prioritize higher-order systems<br>2.2 Prioritize functionality over patterns<br>2.3 Flexible protection<br>2.4 Biomass, diversity and network	Manage for ecosystem functionality and resilience<br>1 Prioritize higher-order systems (ecosystems) and their processes over lower-order systems (species and life communities)<br>2 Prioritize target functionality over representation of target patterns (e.g. specific components such as species or life communities)<br>3 Refrain from static preservation of species/populations in defined areas<br>4 Manage for high levels of biomass, diversity and network.	Ecosystem functionality and resilience can be best enhanced by focussing on whole ecosystems instead of system parts such as species or communities. Ecosystem functionality increases with the amount of biomass harboured in the ecosystem, the degree of information contained and the degree of networking (complex organisation) among the system's elements.	Ecosystems change, but they change even more and faster under climate change. Therefore they need to be as functional as possible to support their properties of self-organization and self-regulation. Ecosystem functionality is thus important for the maintenance of ecosystem resilience and adaptive capacity, which are all essential for facing and dealing with climate change.	Jørgensen (2006)<br>Huntley (2007)<br>Hobson and Ibisch (2010)<br>Freudenberger et al. (2012)<br>Groves et al. (2012)

Principles and criteria short titles (as used in the main article)	Principles and criteria in full notation	Description	Rationale	References
3 Adequate spatial dimension*	Apply adequate spatial dimensions of planning and management	Apply (eco-)regional approaches, consider targets and strategies within a regional context, account for impact of management activities on nearby ecosystems.	Climate change has many impacts biodiversity, some of which occur with large spatial dimensions such as species' and systems' spatial shifts. It is therefore necessary to consider influencing factors and surrounding regions on a broad scale and to increase the functionality of conservation targets (ecosystems) in order to buffer those changes and to account for them. Applying adequate spatial dimensions is therefore essential for effective conservation planning and management under climate change.	Hannah et al. (2002)<br>Ibisch and Kreft (2008)<br>Lawler (2009)<br>Mawdsley et al. (2009)<br>McLeod et al. (2009)
3.1 Functional ecological boundaries	1 Apply eco-regional approaches and delimit spatial management scope according to ecological boundaries of functional size.			
3.2 Continuity and connectedness	2 Select a spatially continuous management scope and avoid disconnection and cluster structure favoring direct connection to nearby conservation sites or functionally relevant ecosystems.			
3.3 Regional context	3 Consider conservation targets within a regional context and let their scale guide the scale of management strategies.			
3.4 Adjacent ecosystems	4 Consider and manage adjacent ecosystems/regions as areas of influence.			
4 Adequate time dimension*	Apply adequate time dimensions of planning and management	Adapt time dimensions of planning and management to the conservation system, apply a long-term perspective (e.g., extended planning horizon, future-oriented strategy design) and especially include future developments in today's considerations.	Most (climate and climate-induced) changes occur over long time periods and need to be addressed early enough but with a far time horizon to ensure success of conservation measures.	Welch (2005)<br>Lawler (2009)<br>Stein et al. (2013)
4.1 Long-term perspective	1 Adopt a long-term perspective in planning and management (>20 years).			
4.2 Future changes	2 Extend strategy design to include future changes in target status.			
4.3 Activities of different terms	3 Beside short-term and medium-term activities, also include long-term activities in planning and implementation.			
4.4 Long-term impact of activities	4 Assess and account for the long-term impact of planned activities.			

Principles and criteria short titles (as used in the main article)	Principles and criteria in full notation	Description	Rationale	References
5 Holistic knowledge management*	Pursue holistic knowledge and non-knowledge management	Integrate diverse forms of knowledge and non-knowledge into planning in a holistic and constructive approach and establish cooperative partnerships with other organizations, institutions and agencies of different disciplines and sectors as well as with other conservation projects/sites for knowledge transfer exchange and development as well as concerted capacity building.	Climate change not only affects biodiversity but also other systems, such as systems of human land use, which might ultimately affect biodiversity. Further, climate change increases the complexity of conservation and other systems and of their interaction; it generates higher rates of uncertainty. For addressing complexity and uncertainty a holistic management of knowledge and non-knowledge is necessary. In order to effectively manage a system it is important to know as much about the system as well as about climate change impacts as possible and to use different sources of knowledge. In order to deal with uncertainty it is equally important to keep track of non-knowledge.	Ibisch et al. (2010)<br>Ibisch et al. (2012b)<br>Kingston et al. (2015)
5.1 Knowledge tracking	1 Constantly keep track and make use of best-available knowledge assessing limits to knowledge, uncertainty and knowledge gaps.			
5.2 Diverse knowledge forms	2 Consider all forms of relevant information including scientific, indigenous and local knowledge and practice.			
5.3 Diverse disciplines	3 Consider all relevant sectors of society and (scientific) disciplines in the planning process			
5.4 Knowledge exchange	4 Cooperation and knowledge exchange with other organizations, institutions, agencies and conservation projects.			
6 Systemic and strategic coherence**	Maintain systemic and strategic coherence and nestedness	Pursue coherence and strategic alignment in management and planning across administrative levels/planning levels and with and spatial planning units on the same level. Acknowledge coherence and nestedness of subsystems as part of the global ecosystem.	Climate change does not only affect a single system but also its subsystems and the superior system, even with different kinds of impact. Those changes of nested or larger systems may then indirectly also impact the system in focus. Hence all system levels need to be considered and their management needs to be aligned.	BRANCH partnership (2007)<br>Ibisch et al. (2012a)
6.1 System interaction	1 Consider interaction with adjacent as well as superordinate and subordinate (eco)systems.			
6.2 Vertical nestedness	2 Align conservation goals and targets of different planning levels (systematic vertical nestedness)			
6.3 Horizontal coherence	3 Align conservation goals, targets and strategies with other planning units of the same level (horizontal alignment).			
6.4 Inter-protected area management	4 Align conservation goals, targets and strategies with adjacent conservation sites (inter-protected area management).			

Principles and criteria short titles (as used in the main article)		Principles and criteria in full notation	Description	Rationale	References
7	Adaptive management*	Apply adaptive management	Practice target setting, strategy development and implementation, monitoring and evaluative learning in a continual, cyclic and iterative process allowing for uncertainty as well as possible errors and their alleviation throughout the process.	Climate change is connected to a high degree of uncertainty and non-knowledge.<br>Due to its iterative and error-friendly character and strong focus on monitoring and feed-back mechanisms adaptive management allows for managing under uncertainty. With adaptive management approaches (climatic) changes can be discovered and integrated into planning early. It allows for in-time adaptation of goals, targets, strategies and actions to increase conservation effectiveness.	Ibisch and Kreft (2008)<br>Baron et al. (2009)<br>Lawler (2009)<br>Lemieux et al. (2010)<br>Mawdsley (2011)
7.1	Iterative planning	1 Adopt an open, cyclic and iterative planning approach with continuous development allowing for flexibility and early action.			
7.2	Systematic monitoring	2 Establish a strong systematic monitoring system aligning it along conservation targets and goals with the aim of measuring goal achievement.			
7.3	Adaptive target and goal setting	3 Adapt the choice of conservation targets and goals as well as management activities to changing conditions (e.g., community changes) and goal achievement.			
7.4	Evaluation of effectiveness	4 Monitor and evaluate success of management effectiveness and create learning feedback loops to adapt management.			
8	Proactive risk management	Apply proactive risk management	Consider potential future developments, risks and vulnerability in goal-setting and strategy development, plan with foresight and alternative options despite non-knowledge and uncertainty applying the precautionary principle and prioritize robust strategies.	Climate change comes with great uncertainties and increases the risk potential for conservation systems, for example due to increased extreme events and higher weather variability. Climate change does not only affect conservation target per se but also other systems such as land use systems, which might increase the risk for conservation systems. Proactive risk management acknowledges that anticipatory rather than reactive approaches to conservation are essential when dealing with climate change. It facilitates the preparation for potential changes through anticipation and risk analysis. This enables adapting strategies before changes really affect a system, not only afterwards, and can save costs and ensure effectiveness.	Willows and Connell (2003)<br>Ibisch and Kreft (2008)<br>Hallegatte (2009)<br>Ibisch et al. (2009)
8.1	Precautionary principle	1 Adopt the precautionary principle.			
8.2	Future target vulnerability	2 Consider (future) risks and vulnerabilities of conservation targets and relevant factors, esp. concerning climate change.			
8.3	Scenario planning	3 Consider plausible futures (scenarios) and trends in goal-setting and strategy development.			
8.4	Robust strategies	4 Identify and prioritize robust (no-and low-regret) strategies (those that will be effective in most scenarios).			

Principles and criteria short titles (as used in the main article)		Principles and criteria in full notation	Description	Rationale	References
9	Institutional capacity building*	Build institutional capacity for functional management and especially for dealing with climate change	Establish functional planning and management structures capable of dealing with climate change and improve internal capacity (knowledge and abilities of management staff).	Only with sufficient (institutional) capacity, especially to deal with climate change but also in general, effective management in the face of climate change is possible.	Welch (2005)<br>Lemieux et al. (2010)<br>Müller et al. (2015)
	9.1 Decentralization and responsibility	1 Decentralize planning and management to the lowest appropriate level and ensure clear responsibilities.			
	9.2 Transdisciplinarity of team	2 Create a transdisciplinary planning and management team with diverse personal resources.			
	9.3 Knowledge and research capacities	3 Professionally train staff for climate change and improve research capacities of conservation staff.			
	9.4 Methodological training	4 Ensure training & knowledge update of relevant methodologies and instruments to face climate change in management.			
10	Public accountability and acceptance	Pursue transparency, public accountability and acceptance	Communicate with and inform the public regularly about the status and development of conservation management and about climate change and its implications for the region. Ensure participation and consideration of stakeholders in management and planning.	Climate change poses a particular challenge to conservation managing systems such as protected areas that in many cases tend to have low management effectiveness even without climate change. In order to successfully deal with aspects of climate change in management it is necessary to guarantee a basic functioning of the conservation management system. The acceptance and the support of the public represent preconditions for effectiveness. Resistance, conflicts and counteraction minimizing opportunities to deal with climate change will hamper management. Further, any effort towards climate change adaptation will be ineffective without public support. Protected areas do not function in isolation but within a local and/or	Welch (2005)<br>Lemieux et al. (2010)<br>Dovers et al. (2015)
	10.1 Participation	1 Involve the public (esp. land users and stakeholders s.str.) in management and planning			
	10.2 Regular public reporting	2 Regularly report on the state of the conservation site and its surroundings, on ongoing and planned management activities and their implications to the public.			
	10.3 Acceptance-increasing strategies	3 Actively increase and ensure acceptance and support through specific strategies (like local product marketing, tourism marketing, monitoring by local population etc.).			
	10.4 Public information	4 Inform the public about climate change and its implications on local, regional and national and international level and about efforts to mitigate and			

Principles and criteria short titles (as used in the main article)	Principles and criteria in full notation	Description	Rationale	References
	adapt to climate change, ecosystem-based adaptation and climate management.		regional system. Therefore, conservation under climate change requires an integrative approach that includes all people in and around protected areas, especially land users. They need to be considered an essential part of (conservation) systems.	
11 Matrix and stakeholder management*<br><br>11.1 Regional context<br><br>11.2 Stakeholder cooperation<br><br>11.3 Concerted strategies<br><br>11.4 Cooperative ecosystem-based climate management	Manage into the matrix (utilized land) and establish cooperative partnerships with local and regional land users and other stakeholders<br><br>1 Manage beyond the borders of the conservation site and within a regional context.<br><br>2 Cooperate with land users and stakeholders in- and outside the site's borders and concert strategies.<br><br>3 Consider current and potential future climate change adaptation activities of land users and stakeholders in and around conservation site.<br><br>4 Engage land users and local stakeholders in cooperation on ecosystem-based climate management.	Manage beyond protected area (official) borders into the matrix balancing utilization and conservation of biodiversity and cooperate with, actively involve and concert strategies with land users and other stakeholders in planning and management, esp. consider their current and potential adaptation to climate change adaptation activities.	Conservation systems are connected with and embedded in other systems such as human (land use or political) systems. Climate change is a global issue and therefore affects all those systems equally. Since most threats and influencing factors on conservation targets occur in the surroundings of conservation sites, those surroundings are important for the connection of individual sites. Therefore, it is essential to pursue an integrative ecosystem management approach to account for (climate) changes in all relevant systems and to support conservation effectiveness.<br>In times of climate change, conservation management is facing the need to consequently and effectively implement strategies that exceed current dimensions and to engage in cooperation with land users and stakeholders much more. It is important to not only acknowledge but also communicate the higher relevance of conservation and climate change to society than traditionally considered.	Hannah et al. (2002)<br>Ibisch and Kreft (2008)<br>Schliep et al. (2008)<br>Lemieux et al. (2010)<br>Lindenmayer et al. (2010)
