# Supplementary material for: Assessing climate change-robustness of protected area management plans—The case of Germany
Source: PLoS One. 2017 Oct 5;12(10):e0185972. doi: 10.1371/journal.pone.0185972 (PMC5628909; doi:10.1371/journal.pone.0185972)
Supplement: S2 Table — (PDF) [file pone.0185972.s003.pdf]

S2 Table. References of the 60 management plans analysed in the study on climate change-robustness of protected area management plans in Germany.

Protected area name[a]	Name of plan[b]	Responsible institution[c]	Version[d]	URL[e]
Biosphärenreservat Flusslandschaft Elbe	Rahmenkonzept für das länderübergreifende UNESCO-Biosphärenreservat „Flusslandschaft Elbe“	Ministerium für Landwirtschaft, Umwelt und Verbraucherschutz Mecklenburg Vorpommern, Stralsund<br>Ministerium für Landwirtschaft und Umwelt des Landes Sachsen-Anhalt, Magdeburg,<br>Ministerium für ländliche Entwicklung, Umwelt und Verbraucherschutz des Landes Brandenburg, Potsdam<br>Niedersächsisches Umweltministerium, Hannover,<br>Ministerium für Landwirtschaft, Umwelt und ländliche Räume des Landes Schleswig-Holstein, Kiel	12/2006	http://www.flusslandschaft-elbe.de/cms/upload/downloads/Rahmenkonzept%20BR%20Flusslandschaft%20Elbe-fertig-April-07.pdf
Biosphärenreservat Karstlandschaft Südharz	Rahmenkonzept für das Biosphärenreservat Karstlandschaft Südharz	Landesverwaltungsamt Sachsen-Anhalt, Biosphärenreservat Karstlandschaft Südharz, Roßla/Südharz	10/2011	
Biosphärenreservat Niedersächsische Elbaue	Biosphärenreservatsplan mit integriertem Umweltericht – Biosphärenreservat Niedersächsische Elbtalaue	Biosphärenreservatsverwaltung Niedersächsische Elbtalaue, Hitzacker	03/2009	https://www.elbtalaue.niedersachsen.de/biosphaerenreservat/biosphaerenreservatsplan/der-biosphaerenreservatsplan-53983.html
Biosphärenreservat Schaalsee	Rahmenkonzept Biosphärenreservat Schaalsee, Band Leitbild und Ziele,<br><br>Rahmenkonzept Biosphärenreservat Schaalsee, Kurzfassung: Leitbild und Ziele	Amt für das Biosphärenreservat Schaalsee, Zarrentin	11/2003	https://www.schaalsee.de/inhalte/download/leitbild.pdf<br><br>https://www.schaalsee.de/inhalte/download/ziele.pdf
Biosphärengebiet Schwäbische Alb	Biosphärengebiet Schwäbische Alb – Das Rahmenkonzept<br>Band 1 Unser Gebiet: Ausgangssituation, Herausforderungen und Entwicklungspotenziale<br>Band 2 Unsere Strategie: Leitbilder, Entwicklungsziele, Leitprojekte (Kurzfassung)<br>Band 3 Unser Weg: Leitprojekte, Projekte	Ministerium für Ländlichen Raum und Verbraucherschutz Baden-Württemberg, Regierungspräsidium Tübingen<br>Geschäftsstelle Biosphärengebiet Schwäbische Alb, Münsingen-Auingen<br>PLENUM Schwäbische Alb, Reutlingen	07/2012	http://biosphaerengebiet-alb.de/images/lebensraum/downloads/Band-1-Unser-Gebiet-24.08.2012-endgltig.pdf<br><br>http://biosphaerengebiet-alb.de/images/lebensraum/downloads/Band-2-Unsere-Strategie-24.08.2012-endgltig.pdf<br><br>http://biosphaerengebiet-

Protected area name[a]	Name of plan[b]	Responsible institution[c]	Version[d]	URL[e]
	und Projektideen			alb.de/images/lebensraum/downloads/Band-3-Unser-Weg-24.08.2012-endgltig.pdf
Biosphärenreservat Vessertal-Thüringer Wald	Grundlagenband zum Rahmenkonzept zur Entwicklung und zum Schutz des Biosphärenreservats Vessertal-Thüringer Wald<br><br>Rahmenkonzept zur Entwicklung und zum Schutz des Biosphärenreservats Vessertal-Thüringer Wald	Verwaltung Biosphärenreservat Vessertal-Thüringer Wald, Schmiedefeld a. R.	11/2006	https://www.biosphaerenreservat-thueringerwald.de/de/service/infomaterialien/rahmenkonzept2006_screen.pdf
DE 6410-301<br>FFH Ackerflur bei Ulmet	NATURA 2000 – Bewirtschaftungsplan für das Fauna-Flora-Habitat-Gebiet "Ackerflur bei Ulmet" 6410-301<br>Teil A: Grundlagen<br>Teil B: Maßnahmen	Struktur- und Genehmigungsdirektion Süd, Neustadt an der Weinstraße, Obere Naturschutzbehörde der SGD Süd	08/2008	http://map.final.rlp.de/docs_kartendienste/BWP_2008_01_S/BWP_2008_01_S_Fachplan_Grundlagen.pdf<br><br>http://map.final.rlp.de/docs_kartendienste/BWP_2008_01_S/BWP_2008_01_S_Fachplan_Maßnahmen.pdf
SPA 8433-401<br>Bayrischer Bodensee	Managementplan für das Natura 2000-Gebiet „Bayrischer Bodensee" 8433-401	Regierung von Schwaben, Sachgebiet 51, höhere Naturschutzbehörde, Augsburg	11/2012	Neue Version (2014):<br>http://www.regierung.schwaben.bayern.de/Aufgaben/Bereich_5/Naturschutz_und_Landschaftspflege/Nat-Bodensee/Text_MPl_BayerBodensee_Entwurf_September2014.pdf
DE-2123-301<br>FFH Binnendünen Nordoe	Managementplan für das Fauna-Flora-Habitat-Gebiet DE-2123-301 „Binnendünen Nordoe" (Bürgerfassung)	Ministerium für Landwirtschaft, Umwelt und ländliche Räume des Landes Schleswig – Holstein, Kiel	05/2011	http://www.kremperheide.de/daten/dateien/allgemeines/FFH_MP_Nordoe_101118.pdf
DE 2918-302<br>FFH Rethriehen	Managementplan FFG-Gebiet Retriehen DE 2918-302	Senator für Bau, Umwelt und Verkehr – Naturschutzbehörde, Bremen	12/2006	https://www.google.de/url?sa=t&rct=j&q=&esrc=s&source=web&cd=1&ved=0ahUKEwinzcX4p7jVAhUCYVAKHQ9RBtsQFggmMAA&url=http%3A%2F%2Fwww.bauumwelt.bremen.de%2Fsixcms%2Fmedia.php%2F13%2FMP%2520FFH-Rethriehen%252012-2006b.pdf&usg=AFQjCNETYCK2Q_f212wCeeY8JypnBKRf9A
DE 2750-302<br>FFH Blumberger Wald	Managementplanung Natura 2000 in im Land Brandenburg. Managementplan für das Gebiet „Blumberger Wald" DE 2750-302	Ministerium für Umwelt, Gesundheit und Verbraucherschutz des Landes Brandenburg (MUGV), Potsdam<br>Stiftung Naturschutzfonds Brandenburg, Potsdam	05/2012	http://www.mlul.brandenburg.de/n/natura2000/managementplanung/blumberger_wald/020_mp_langfassung.pdf

Protected area name[a]	Name of plan[b]	Responsible institution[c]	Version[d]	URL[e]
DE 4634-301 FFH Borntal, Feuchtgebiet und Heide bei Allstedt incl. Erweiterungsflächen	Managementplan für das Besondere Schutzgebiet nach FFH-Richtlinie pSCI Nr. 135 „Borntal, Feuchtgebiet und Heide bei Allstedt incl. Erweiterungsflächen“	Ministerium für Landwirtschaft und Umwelt des Landes Sachsen-Anhalt	01/2006	New version: https://lau.sachsen-anhalt.de/naturschutz/natura-2000/managementplanung/borntal-feuchtgebiet-und-heide-bei-allstedt-incl-erweiterungsflaechen/
DE 3751-302 FFH Drahendorfer Spreeniederung	Managementplanung Natura 2000 in im Land Brandenburg. Managementplan für das Gebiet „Drahendorfer Spreeniederung“ DE 3751-302	Ministerium für Umwelt, Gesundheit und Verbraucherschutz des Landes Brandenburg (MUGV), Potsdam<br>Stiftung Naturschutzfonds Brandenburg, Potsdam	08/2012	http://www.mlul.brandenburg.de/n/natura2000/managementplanung/560/mp560_lang.pdf
DE 8226-341 FFH Feuchtgebietskomplexe nördlich Isny	Managementplan für das FFH-Gebiet 8226-341 Feuchtgebietskomplexe nördlich Isny	Regierungspräsidium Tübingen, Referat 56 – Naturschutz und Landschaftspflege	01/2011	https://www4.lubw.baden-wuerttemberg.de/servlet/is/207772/8226341_Feuchtgebietskomplexe_noerdlich_Isny/8226341_02_text_managementplan.pdf?command=downloadContent&filename=8226341_Feuchtgebietskomplexe_noerdlich_Isny/8226341_02_text_managementplan.pdf
DE 5144-301 FFH Flöhatal	Managementplan für das FFH-Gebiet 5144-301 „Flöhatal“ (Landkreis Freiberg und Mittlerer Erzgebirgskreis) – Endbericht	Regierungspräsidium Chemnitz	11/2005	
DE 2117-301 FFH Heide und Heideweiher auf der Rekumer Geest	Pflege- und Managementplan (PMP) für das FFH-Gebiet "Heide und Heideweiher auf der Rekumer Geest" 2011	Der Senator für Umwelt, Bau, Verkehr und Europa, Bremen<br>SUBVE Naturschutzbehörde Bremen	04/2011	https://www.google.de/url?sa=t&rct=j&q=&esrc=s&source=web&cd=1&ved=0ahUKEwiczM3asLjVAhWCUlAKHS0XDTUQFggmMA&url=http%3A%2F%2Fwww.bauumwelt.bremen.de%2Fsixcms%2Fmedia.php%2F13%2FPMP%2520Heide-Weiher_2011_Text.pdf&usg=AFQjCNFXS6CnnqUT4lvui50osF-5okBJVQ
DE 6116-350/ 6116-450 FFH/SPA Kühkopf-Knoblochsaue	Maßnahmenplan (Bewirtschaftungsplan) für das FFH/VS-Gebiet 6116-350/ 6116-450 "Kühkopf-Knoblochsaue"	Regierungspräsidium Darmstadt, Hess-Forst Forstamt Groß-Gerau	01/2011	https://schatzinsel-kuehkopf.hessen.de/sites/sikk.hessen.de/files/content-downloads/Ma%C3%9Fnahmenplan%20FFH%20VS-Gebiet%20K%C3%BChkopf-Knoblochsaue.pdf
DE 5322-306 FFH Lauter und Eisenbach	Maßnahmenplan für das FFH- Gebiet DE 5322-306 „Lauter und Eisenbach“	Regierungspräsidium Giessen, Kreis Vogelsberg, Forstamt Romrod	2012	https://www.lautertal-vogelsberg.de/gemeinde/wohnen-leben/naturschutz/ffh-gebiete.html

Protected area name[a]	Name of plan[b]	Responsible institution[c]	Version[d]	URL[e]
DE6717-341<br>FFH Lußhardt zwischen Reilingen und Karlsdorf<br>DE6817-441<br>SPA Saalbachniederung bei Hambrücken<br>DE 6916-441<br>SPA Hardtwald nördlich von Karlsruhe (Erweiterung)	Managementplan für die Natura 2000-Gebiete 6717-341 „Lußhardt zwischen Reilingen und Karlsdorf“ 6817-441 „Saalbachniederung bei Hambrücken“ 6916-441 „Hardtwald nördlich von Karlsruhe (Erweiterung)“	Regierungspräsidium Karlsruhe, Referat 56 – Naturschutz und Landschaftspflege	11/2012	http://www4.lubw.baden-wuerttemberg.de/servlet/is/223274/
DE 6419-301<br>FFH Magerwiesen Raubach	Maßnahmenplan für das FFH- Gebiet Magerwiesen von Raubach	Regierungspräsidium Darmstadt, Odenwaldkreis - Amt für den ländlichen Raum, Reichelsheim	05/2009	
DE 3536-301<br>FFH/SPA Mahlpfuhler Fenn	Managementplan für das FFH-Gebiet (SCI) 35 und das Vogelschutzgebiet (SPA) 26 „Mahlpfuhler Fenn“<br>FFH_0035 (DE 3536-301) und SPA_0026 (DE 3536-301)	Landesamt für Umweltschutz Sachsen-Anhalt FB 4, Halle	04/2012	https://lau.sachsen-anhalt.de/fileadmin/Bibliothek/Politik_und_Verwaltung/MLU/LAU/Naturschutz/Natura2000/Managementplanung/Dateien/Mahlpfuhler_Fenn_Text.pdf
DE 6205 – 303<br>FFH Mattheiser Wald	NATURA 2000: Bewirtschaftungsplan für das FFH-Gebiet Mattheiser Wald Gebietsnummer 6205 – 303<br>Teil A: Grundlagen<br>Teil B: Maßnahmen	Struktur- und Genehmigungsdirektion Nord Obere Naturschutzbehörde, Koblenz	2010	http://map.final.rlp.de/docs_kartendienste/BWP_2010_02_N/BWP_2010_02_N_Fachplan_Grundlagen.pdf<br><br>http://map.final.rlp.de/docs_kartendienste/BWP_2010_02_N/BWP_2010_02_N_Fachplan_Maßnahmen.pdf
DE 8028-371<br>FFH Mindelquellgebiet	Managementplan für das FFH-Gebiet 8028-371 „Mindelquellgebiet“	Regierung von Schwaben, Sachgebiet 51 – höhere Naturschutzbehörde, Augsburg	12/2012	
DE-1631-393<br>FFH Nordseite der Wagrischen Halbinsel	Managementplan für das Fauna-Flora-Habitat-Gebiet DE-1631-393 „Nordseite der Wagrischen Halbinsel“ und das Europäische Vogelschutzgebiet DE-1530-491 „Östliche Kieler Bucht“ Teilgebiet: FFH-Gebiet DE-1631-393	Ministerium für Landwirtschaft, Umwelt und ländliche Räume des Landes Schleswig-Holstein, Kiel	06/2012	http://www.heiligenhafen.de/fileadmin/download/1631_mplan_120423.pdf
DE 5337-301<br>SCI Nordwestvogt-ländische Teiche	Managementplan für das SCI 296 / DE 5337-301 „Nordwestvogtländische Teiche und Moor Oberlinda“ (Vogtlandkreis)	Sächsisches Landesamt für Umwelt, Landwirtschaft und Geologie (LfULG), Dresden	11/2010	

Protected area name[a]	Name of plan[b]	Responsible institution[c]	Version[d]	URL[e]
DE 8214-343<br>FFH Oberer Hotzenwald	Managementplan für das FFH-Gebiet 8214-343 "Oberer Hotzenwald" und das Vogelschutzgebiet 8114 401"Südschwarzwald" (Teilgebiet Oberer Hotzenwald) Maßnahme A.2 des LIFE-Natur-Projekts Oberer Hotzenwald	Regierungspräsidium Freiburg, Referat 56 - Naturschutz<br>und Landschaftspflege, Freiburg	10/2010	http://www4.lubw.baden-wuerttemberg.de/servlet/is/73083/
DE-2330-353<br>FFH/SPANSG<br>Oldenburger See und Umgebung	Managementplan für das Fauna-Flora-Habitat-Gebiet / EU-Vogelschutzgebiet DE-2330-353 „NSG Oldenburger See und Umgebung"	Ministerium für Landwirtschaft, Umwelt und ländliche Räume des Landes Schleswig-Holstein, Kiel	11/2010	http://www.umweltdaten.landsh.de/public/natura/pdf/mplan_inet/2330-353/2330-353MPlan_Text.pdf
DE 5905-401<br>SPA Orsfeld	NATURA 2000 - Bewirtschaftungsplan für das Vogelschutzgebiet „Orsfeld (Bitburger Gutland)"<br>Gebietsnummer 5905-401<br>Teil A: Grundlagen<br>Teil B: Maßnahmen	Struktur- und Genehmigungsdirektion Nord Rheinland-Pfalz, Koblenz	2009	http://map.final.rlp.de/docs_kartendienste/BWP_2009_03_N/BWP_2009_03_N_Fachplan_Grundlagen.pdf<br><br>http://map.final.rlp.de/docs_kartendienste/BWP_2009_03_N/BWP_2009_03_N_Fachplan_Maßnahmen.pdf
DE 4639-303<br>FFH Pfeifengraswiese bei Günthersdorf	Managementplan für das FFH-Gebiet „Pfeifengraswiese bei Günthersdorf"<br>FFH_0283 (DE 4639-303)	Landesamt für Umweltschutz Sachsen-Anhalt, Fachbereich 4, Halle	02/2011	https://lau.sachsen-anhalt.de/fileadmin/Bibliothek/Politik_und_Verwaltung/MLU/LAU/Naturschutz/Natura2000/Managementplanung/Dateien/Pfeifengraswiese-bei-Guenthersdorf_ges.pdf
DE 2650 -301<br>FFH Randowhänge bei Schmölln	Managementplanung Natura 2000 im Land Brandenburg - Managementplan für das Gebiet „Randowhänge bei Schmölln"<br>Landesinterne Melde Nr. 456, EU-Nr. DE 2650 – 301	Ministerium für Umwelt, Gesundheit und Verbraucherschutz des Landes Brandenburg (MUGV), Potsdam<br>Stiftung Naturschutzfonds Brandenburg, Potsdam	05/2012	http://www.mlul.brandenburg.de/n/natura2000/managementplanung/randowhaenge/mp456.pdf
DE 2750-306<br>FFH Randowtal bei Grünz und Schwarze Berge	Managementplan für das FFH-Gebiet DE 2750-306 „Randowtal bei Grünz und Schwarze Berge"	Staatliches Amt für Landwirtschaft und Umwelt Vorpommern, Stralsund	10/2011	http://www.stalu-mv.de/vp/Themen/Naturschutz-und-Landschaftspflege/Natura-2000/Managementplanung/DE-2750-306-Randowtal-bei-Gruenz-und-Schwarze-Berge
DE 2750 -301<br>FFH Randow-Welse-Bruch	Managementplanung Natura 2000 im Land Brandenburg - Managementplan für das Gebiet „Randow-Welse-Bruch"<br>Landesinterne Melde Nr. 460, EU-Nr. DE 2750 –301	Ministerium für Umwelt, Gesundheit und Verbraucherschutz des Landes Brandenburg (MUGV), Potsdam<br>Stiftung Naturschutzfonds Brandenburg, Potsdam	05/2012	http://www.mlul.brandenburg.de/n/natura2000/managementplanung/randow_welse_bruch/460_mp_langfassung.pdf
DE 6308-304<br>SPA Rothenberg	Natura 2000-Managementplanung Saarland 2010, Vogelschutzgebiet	Ministerium für Umwelt und Verbraucherschutz Saarland, Saarbrücken	05/2013	

Protected area name[a]	Name of plan[b]	Responsible institution[c]	Version[d]	URL[e]
	„Rothenberg“ 6308-304			
DE 5807-401<br>SPA Sangweiher	Natura 2000 – Bewirtschaftungsplan für das Vogelschutzgebiet „NSG Sangweiher und Erweiterung“ (5807-401)<br>Teil A: Grundlagen<br>Teil B: Maßnahmen	Struktur- und Genehmigungsdirektion Nord Rheinland-Pfalz, Koblenz	2011	http://map.final.rlp.de/docs_kartendienste/BWP_2011_24_N/BWP_2011_24_N_Fachplan_Grundlagen.pdf<br><br>http://map.final.rlp.de/docs_kartendienste/BWP_2011_24_N/BWP_2011_24_N_Fachplan_Maßnahmen.pdf
DE 5051-301<br>SCI Sebnitzer Wald	Managementplan für das SCI 165 „Sebnitzer Wald und Kaiserberg“	Staatsbetrieb Sachsenforst, Obere Forst- und Jagdbehörde, Pirna/Graupa	03/2011	
DE 6833-371<br>FFH Trauf der südlichen Frankenalb	Managementplan für das FFH-Gebiet 6833-371 »Trauf der südlichen Frankenalb«<br>Fachgrundlagen<br>Maßnahmen	Amt für Ernährung, Landwirtschaft und Forsten<br>Ansbach, Bayern, Natura 2000-Regionalteam Mittelfranken; Dinkelsbühl	05/2011<br>09/2010	
DE-4203-401<br>SPA Unterer Niederrhein	Maßnahmenkonzept für das EU Vogelschutzgebiet „Unterer Niederrhein“ DE-4203-401	Ministerium für Klimaschutz, Umwelt, Landwirtschaft, Natur- und Verbraucherschutz NRW (MKULNV), Düsseldorf, Landesamt für Natur, Umwelt und Verbraucherschutz NRW (LANUV)	02/2011	https://www.lanuv.nrw.de/fileadmin/lanuv/natur/schutzgeb/vogelschutzgebiete/mako/MAKO_VSG_Unterer_Niederrhein_Endfassung.pdf
DE 2138-302<br>FFH Warnowtal	Managementplan für das FFH-Gebiet DE 2138-302 Warnowtal mit Zuflüssen<br>Teilgebiet: Nördlicher Teilbereich	Staatliches Amt für Landwirtschaft und Umwelt Mittleres Mecklenburg, Abteilung Naturschutz, Wasser und Boden; Rostock	11/2011	http://www.stalu-mv.de/wm/Themen/Naturschutz-und-Landschaftspflege/NATURA-2000/FFH-Managementplanung/DE-2138-302-Warnowtal-mit-kleinen-Zufluessen
DE 1934-302<br>FFH Wismarbucht	Managementplan für das FFH-Gebiet DE 1934-302 Wismarbucht (gleichzeitig teilweise Vogelschutzgebiet DE 2043-401 gemäß Vogelschutz-Richtlinie	Umweltministerium Mecklenburg-Vorpommern, Referat Landschaftsplanung, Management der Natura 2000 Gebiete, Schwerin	02/2006	http://ec.europa.eu/ourcoast/download.cfm?fileID=1187
Nationalpark Bayrischer Wald	Nationalparkplan<br>Hauptband: Leitbild und Ziele<br>Anlageband Wegeplan<br>Anlageband Walderhaltungs- und Waldpflegemaßnahmen<br>Anlageband. Schalenwildmanagement<br>Anlageband Renaturierung<br>Anlageband Nutzungen und Gestattungen	Nationalparkverwaltung Bayrischer Wald, Grafenau	2010	http://www.nationalpark-bayerischer-wald.de/wir_ueber_uns/rechtliche_grundlagen/nationalparkplan/index.htm

Protected area name[a]	Name of plan[b]	Responsible institution[c]	Version[d]	URL[e]
	Anlageband Forschung<br>Anlageband Bildungs-und Erholungseinrichtungen<br>Anlageband Arten- und Biotopschutz<br>Anlageband Bildungs- Informations- und Öffentlichkeitsarbeit			
Nationalpark Hainich	Nationalparkplan für den Nationalpark Hainich - Leitbild und Ziele	Nationalpark Hainich, Bad Langensalza	02/2010	http://www.nationalpark-hainich.de/fileadmin/Medien/Downloads/NLP-Plan2010_Endfassung.pdf
Nationalpark Harz	Nationalparkplan für den Nationalpark Harz 2011-2020	Nationalparkverwaltung Harz, Wernigerode	02/2011	http://www.nationalpark-harz.de/de/downloads/gesetzliche_grundlagen/Nationalparkplan2011_Internet_Karten.pdf
Nationalpark Kellerwald-Edersee	Nationalparkplan für den Nationalpark Kellerwald-Edersee	Nationalparkamt Kellerwald-Edersee, Bad Wildungen	12/2008	https://www.nationalpark-kellerwald-edersee.de/de/service/downloads/nationalparkplan/downloads/NLP_Plan_2008.pdf
Nationalpark Müritz	Nationalparkplan<br>1) Leitbild und Ziele<br>2) Bestandsanalyse<br>3) Projektübersicht	Landesamt für Forsten und Großschutzgebiete Mecklenburg-Vorpommern, Malchin; Nationalparkamt Müritz, Hohenzieritz	12/2003	http://www.mueritz-nationalpark.de/wissen-und-verstehen/Nationalpark/Nationalparkplan/
Nationalpark Sächsische Schweiz	Nationalpark-Programm für den Nationalpark Sächsische Schweiz	Nationalpark Sächsische Schweiz, Bad Schandau	08/2007	New Version:<br>http://www.nationalpark-saechsische-schweiz.de/wp-content/uploads/2014/01/Nationalparkprogramm-2015.pdf
Nationalpark Unteres Odertal	Nationalparkplan<br>Band 1 Leitbild und Ziele<br>Band 2 Bestandsanalyse<br>Band 3 Projekte und Maßnahmen	Nationalpark Unteres Odertal – Verwaltung, Schwedt/Oder	11/2013	New Version:<br>http://www.nationalpark-unteres-odertal.eu/management/nationalparkplan/
Naturpark Am Stettiner Haff	Naturparkplan Naturpark Am Stettiner Haff<br><br>Band I Leitbild, Ziele und Umsetzungsstrategien<br>Band II Daten und Fakten<br>Band III Projekte	Landesamt für Umwelt, Naturschutz und Geologie, Güstrow<br>Landkreis Uecker-Randow, Pasewalk	11/2008	http://www.google.de/url?sa=t&rct=j&q=&esrc=s&source=web&cd=2&cad=rja&uact=8&ved=0ahUKEwjp_7e76rXVAhXEhrQKHce2BDQQFggsMAE&url=http%3A%2F%2Fresources.natur-mv.de%2Fresource.aspx%2F1fdd50a8-b87d-4456-d5d4-fc910b59f1c2%2FNPPl_ASH_Band_I.pdf&usg=AFQjCNG7Q5-Zx-mJRVN_1OnyHijKMvmyYA<br><br>http://www.google.de/url?sa=t&rct=j&q=&esrc=s&source=web&cd=3&ved=0ahUKEwjp_7e76rXVAhXEhrQKHce2BDQQFgguMAI&

Protected area name[a]	Name of plan[b]	Responsible institution[c]	Version[d]	URL[e]
				url=http%3A%2F%2Fresources.natur-mv.de%2Fresource.aspx%2F3806b9eb-0016-5331-4450-1b2b278f5023%2FNPPl_ASH_Band_II.pdf&usg=AFQjCNFXYNsmiRagpqiIBdhRTqNkexo3WA<br><br>http://www.google.de/url?sa=t&rct=j&q=&esrc=s&source=web&cd=1&ved=0ahUKEwjAj8Oi67XVAhUNb1AKHYgIB4cQFggmMAA&url=http%3A%2F%2Fresources.natur-mv.de%2Fresource.aspx%2Fa9a00f7e-1b0c-90c1-d7a7-cc459fbdd377%2FBand%2520III.pdf&usg=AFQjCNEp6w1jXaOAQKhRncnLruH2VFzzjg
Naturpark Barnim	Pflege- und Entwicklungsplan für den Naturpark Barnim (Kurzfassung)	Senatsverwaltung für Stadtentwicklung Berlin	07/2009	http://www.lfu.brandenburg.de/cms/media.php/lbm1.a.3310.de/pep_barnim.pdf
Naturpark Bayrischer Wald	Pflege- und Entwicklungsplan Naturpark Bayrischer Wald, Teilfortschreibung 2007	Naturpark Bayrischer Wald e. V., Zwiesel	12/2009	http://www.naturpark-bayer-wald.de/files/upload/Naturpark/Verein%20-%20Foerderung/pflege-und-entwicklungsplan_naturpark%20bayerischer%20wald_2009.pdf
Naturpark Dübener Heide	Naturpark Dübener Heide. Pflege- und Entwicklungskonzept. Teil Sachsen-Anhalt.<br>Teil 1: Bestandsanalyse<br>Teil 2: Leitbild und Ziele	Verein Dübener Heide e.V., Pressel	04/2006	http://www.naturpark-duebener-heide.com/dh/deutsch/05_heimat_natur/06_verwaltung/pek_anhalt/deckblatt_teil1.pdf<br><br>http://www.naturpark-duebener-heide.com/dh/deutsch/05_heimat_natur/06_verwaltung/pek_anhalt/teil2.pdf
Naturpark Habichtswald	Studie zur Evaluierung von Projektideen für den Naturpark Habichtswald	Zweckverband Naturpark Habichtswald, Kassel	10/2012	http://www.naturpark-habichtswald.de/fileadmin/Downloads/NPHW-Endbericht-Endfassung-12_2012-Internet.pdf
Naturpark Hüttener Berge	Naturparkplan Hüttener Berge	Naturparkverein Hüttener Berge e. V. Dassau<br>Kreis Rendsburg-Eckernförde, Rendsburg	04/2010	http://www.naturpark-huettenerberge.de/fileadmin/Download/Naturparkplan/Naturparkplan_Endfassung.pdf
Naturpark Schlaubetal	Der Pflege- und Entwicklungsplan für den Naturpark Schlaubetal – Kurzfassung	Landesumweltamt Brandenburg - Abteilung Raumentwicklung/Großschutzgebiete, Naturpark Schlaubetal, Neuzelle	2004	http://www.lugv.brandenburg.de/cms/media.php/lbm1.a.3310.de/pep_stal.pdf
Naturpark Schlei	Naturparkplan Naturpark Schlei	Naturpark Schlei e.V., Kappeln	04/2010	http://www.naturparkschlei.de/media/PDF/Naturpark/NPS-Naturparkplan.pdf

Protected area name[a]	Name of plan[b]	Responsible institution[c]	Version[d]	URL[e]
Naturpark Soonwald-Nahe	Naturparkplan Soonwald-Nahe	Trägerverein Naturpark Soonwald-Nahe e.V.	03/2008	http://www.soonwald-nahe.de/media/download/Naturparkplan_März%202008.pdf
Naturpark Stromberg-Heuchelberg	Naturparkplan Naturpark Stromberg-Heuchelberg	Naturparkzentrum Naturpark Stromberg-Heuchelberg, Zaberfeld	05/2010	http://www.naturpark-stromberg-heuchelberg.de/userfiles/fileadmin-sh/NaturparkplanStromberg-Heuchelberg2010e.pdf
Naturpark Südharz	Naturparkplan Naturpark Südharz EKP Energie-Klima-Plan GmbH,	Naturpark Südharz, Südharzer Tourismusverband e.V. ; Nordhausen	11/2012	http://www.naturpark-suedharz.de/wp-content/uploads/2014/01/Naturparkplan-NP-S%C3%BCdharz.pdf
Naturpark Teutoburgerwald	Masterplan Teutoburger Wald/Eggegebirge	Zweckverband Naturpark Eggegebirge und südlicher Teutoburger Wald, Detmold	02/2010	https://www.naturpark-teutoburgerwald.de/wp-content/uploads/2016/10/NP_TWEG_Masterplan_K21_20100324_einseitig.pdf
Naturpark Unteres Saaletal	Pflege- und Entwicklungskonzeption. Für den Naturpark „Unteres Saaletal“	Verband Naturpark „Unteres Saaletal“ e.V.	2007	http://naturpark.unteres-saaletal.de/files/5814/8706/3223/Pflege_und_Entwicklungskonzeption-Kurzfassung.pdf
Naturpark Zittauer Gebirge	Pflege- und Entwicklungskonzeption Naturpark Zittauer Gebirge - Abschlussbericht	Landkreis Görlitz, Naturpark Zittauer Gebirge; Zittau	06/2011	http://www.naturschutzzentrum-zittau.de/fileadmin/user_pic/fotos/Naturpark/Naturpark-PEK_Abschlussbericht_Juni2011.pdf

[a] Biosphärenreservat, Biosphärengebiet= Biosphere reserve, Nationalpark= National park, Naturpark= Natur park, FFH=Natura 2000 area of the Fauna-Flora-Habitat Directive, SPA=Special Protection Area of Natura 2000 network of the Birds Directive.

[b] All plans only available in German language.

[c] In most cases the responsible institutions publish the plans, however plans are often written by or in cooperation with external contractors.

[d] In some cases an earlier version than the currently valid one was used for the analysis, i.e. one that was available at that time.

[e] URL can only be provided for currently publically accessible plans. Plans without URL are not publically available (anymore) and must be requested from the responsible institution.
