# Supplementary material for: Assessing climate change-robustness of protected area management plans—The case of Germany
Source: PLoS One. 2017 Oct 5;12(10):e0185972. doi: 10.1371/journal.pone.0185972 (PMC5628909; doi:10.1371/journal.pone.0185972)
Supplement: S4 Table — (PDF) [file pone.0185972.s005.pdf]

S4 Table. Breadth, depth and total quality scores for the 11 principles and 44 criteria of the climate change-robustness index.

Principles and criteria			Criteria breadth		Criteria depth		Total quality	
			Score	Rank	Score	Rank	Score	Rank
1	Addressing climate change		0.32		0.23		0.55	
	1.1	Climate change in situation analysis	0,17	31	0,65	10	0,82	26
	1.2	Climate change in goal setting	0,12	35	0,50	29	0,62	37
	1.3	Climate change in strategies	0,15	34	0,50	29	0,65	36
	1.4	Climate change in monitoring and research	0,17	31	0,50	29	0,67	34
2	Ecosystem functionality and resilience		0.65		0.37		1.02	
	2.1	Prioritize higher-order systems	0,38	19	0,65	8	1,04	17
	2.2	Prioritize functionality over patterns	0,35	20	0,64	11	0,99	20
	2.3	Flexible protection	0,32	23	0,55	23	0,87	23
	2.4	Biomass diversity and network	0,45	17	0,69	2	1,14	12
3	Adequate spatial dimension		0.72		0.41		1.12	
	3.1	Functional ecological boundaries	0,48	11	0,59	20	1,07	16
	3.2	Continuity and connectedness	0,60	6	0,67	4	1,27	5
	3.3	Regional context	0,33	21	0,55	25	0,88	22
	3.4	Adjacent ecosystems	0,47	14	0,64	11	1,11	13
4	Adequate time dimension		0.42		0.24		0.65	
	4.1	Long-term perspective	0,32	23	0,53	26	0,84	25
	4.2	Future changes	0,03	41	0,50	29	0,53	41
	4.3	Activities of different terms	0,32	23	0,50	29	0,82	26
	4.4	Long-term impact of activities	0,10	36	0,50	29	0,60	38
5	Holistic knowledge management		0.90		0.39		1.29	
	5.1	Knowledge tracking	0,65	4	0,51	28	1,16	10
	5.2	Diverse knowledge forms	0,70	2	0,62	16	1,32	3
	5.3	Diverse disciplines	0,53	9	0,66	6	1,19	8
	5.4	Knowledge exchange	0,47	14	0,63	15	1,09	15
6	Systemic and strategic coherence		0.82		0.34		1.16	
	6.1	System interaction	0,58	7	0,60	17	1,18	9
	6.2	Vertical nestedness	0,65	4	0,65	7	1,30	4
	6.3	Horizontal coherence	0,45	17	0,56	22	1,01	19
	6.4	Inter-protected area management	0,20	28	0,50	29	0,70	29
7	Adaptive management		0.63		0.26		0.89	
	7.1	Iterative planning	0,22	27	0,50	29	0,72	28
	7.2	Systematic monitoring	0,48	11	0,55	24	1,04	18
	7.3	Adaptive target and goal setting	0,20	28	0,50	29	0,70	29
	7.4	Evaluation of effectiveness	0,33	21	0,53	27	0,86	24
8	Proactive risk management		0.30		0.18		0.48	
	8.1	Precautionary principle	0,18	30	0,50	29	0,68	31
	8.2	Future target vulnerability	0,17	31	0,50	29	0,67	34
	8.3	Scenario planning	0,03	41	0,50	29	0,53	41
	8.4	Robust strategies	0,05	40	0,50	29	0,55	40
9	Institutional capacity building		0.58		0.24		0.82	
	9.1	Decentralisation and responsibility	0,55	8	0,65	9	1,20	7
	9.2	Transdisciplinarity of team	0,30	26	0,64	13	0,94	21
	9.3	Knowledge and research capacities	0,00	43	0,00	43	0,00	43
	9.4	Methodological training	0,00	43	0,00	43	0,00	43
10	Public accountability and acceptance		0.85		0.35		1.20	
	10.1	Participation	0,73	1	0,64	14	1,37	1
	10.2	Regular public reporting	0,50	10	0,60	17	1,10	14
	10.3	Acceptance-increasing strategies	0,47	14	0,75	1	1,22	6
	10.4	Public information	0,10	36	0,58	21	0,68	31
11	Matrix and stakeholder management		0.75		0.29		1.04	
	11.1	Regional context	0,48	11	0,67	3	1,16	11
	11.2	Stakeholder cooperation	0,68	3	0,66	5	1,34	2
	11.3	Concerted strategies	0,08	38	0,50	29	0,58	39
	11.4	Cooperative ecosystem-based climate management	0,08	38	0,60	17	0,68	31
